# Artificial intelligence fully automated analysis of handheld echocardiography in real‐world patients with suspected heart failure

**DOI:** 10.1002/ejhf.3783

**Published:** 2025-07-24

**Authors:** Ross T. Campbell, Mark C. Petrie, Kieran F. Docherty, Katriona J.M. Brooksbank, Gemma McKinley, Caroline Haig, Alex McConnachie, Carolyn S.P. Lam, Carly Adamson, Elaine Butler, James P. Curtain, Nick Hartshorne‐Evans, Fraser J. Graham, Helen Hainey, John Jarvie, Matthew M.Y. Lee, Leeanne Macklin, Kenneth Mangion, Aimee McCoubrey, Kirsty McDowell, Aileen McIntyre, Sabrina Nordin, Joanna Osmanska, Pierpaolo Pellicori, Joanne Simpson, Piotr Sonecki, Karen Taylor, Daniel Taylor‐Sweet, Pamela Turnbull, Paul Welsh, John J.V. McMurray, Clare L. Murphy, David J. Lowe

**Affiliations:** ^1^ School of Cardiovascular and Metabolic Health University of Glasgow Glasgow UK; ^2^ Robertson Centre for Biostatistics School of Health and Wellbeing, University of Glasgow Glasgow UK; ^3^ National Heart Centre Singapore and Duke‐National University of Singapore Singapore Singapore; ^4^ Pumping Marvellous Preston UK; ^5^ Cardiology Department Queen Elizabeth University Hospital Glasgow UK; ^6^ Cardiology Department Forth Valley Royal Hospital Larbert UK; ^7^ Research and Innovation department NHS Greater Glasgow and Clyde Glasgow UK; ^8^ Cardiology Department Glasgow Royal Infirmary Glasgow UK; ^9^ School of Health and Wellbeing University of Glasgow Glasgow UK; ^10^ Emergency Department Queen Elizabeth University Hospital Glasgow UK

**Keywords:** Heart failure, Diagnosis, Artificial intelligence, Echocardiography, Point of care, Handheld

## Abstract

**Aims:**

Echocardiography is a rate‐limiting step in the timely diagnosis of heart failure (HF). Automated reporting of echocardiograms has the potential to streamline workflow. The aim of this study was to test the diagnostic accuracy of fully automated artificial intelligence (AI) analysis of images acquired using handheld echocardiography and its interchangeability with expert human‐analysed cart‐based echocardiograms in a real‐world cohort with suspected HF.

**Methods and results:**

In this multicentre, prospective, observational study, patients with suspected HF had two echocardiograms: one handheld portable and one cart‐based scan. Both echocardiograms were analysed using fully automated AI software and by human expert sonographers. The primary endpoint was the diagnostic accuracy of AI‐automated analysis of handheld echocardiography to detect left ventricular ejection fraction (LVEF) ≤40%. Other endpoints included the interchangeability (assessed using individual equivalence coefficient [IEC]), between AI‐automated and human analysis of cart‐based LVEF. A total of 867 patients participated. The AI‐automated analysis produced an LVEF in 61% of the handheld scans and 77% of the cart‐based scans, compared to 76% and 77% of human analyses of the handheld and cart‐based scans, respectively. The AI‐automated analysis of handheld echocardiography had a diagnostic accuracy of 0.93 (95% confidence interval [CI] 0.90, 0.95) for identifying LVEF ≤40% (compared to the human analysis of cart‐based transthoracic echocardiography scans). AI‐automated analysis of LVEF on handheld devices was interchangeable with cart‐based LVEF reported by two expert humans (IEC −0.40, 95% CI −0.60, −0.16).

**Conclusions:**

Artificial intelligence‐automated analysis of handheld echocardiography had good diagnostic accuracy for detecting LVEF ≤40%. AI‐automated analysis of LVEF of handheld scans was interchangeable with cart‐based expert human analysis.

## Introduction

Transthoracic echocardiography (TTE) is the guideline‐recommended diagnostic test for heart failure (HF) and guides the initiation of disease‐modifying and prognostic therapies based on left ventricular ejection fraction (LVEF).^1^ In patients presenting in the community with suspected HF, a delayed diagnosis is associated with worse outcomes including hospitalization due to HF and increased mortality.^2^ The main rate‐limiting step in the timely diagnosis of HF is access to TTE. Delays in TTE are often driven by a supply–demand mismatch, with the number of referrals overwhelming the ability of many healthcare systems to provide this important diagnostic test in a timely fashion. The demand for TTE is likely to worsen with ageing populations and the increasing prevalence of HF.^3^

Transthoracic echocardiography is a complex diagnostic examination which takes an expert approximately 45 minutes to acquire and interpret images, usually in a secondary care setting.^4^ Artificial intelligence (AI) algorithms which perform fully automated analysis of TTE images are as accurate as the measurements made by human experts and could accelerate workflow and increase capacity when incorporated into a HF diagnostic pathway.^5^, ^6^ The combination of automated AI analysis and portable handheld TTE devices has the potential to increase the availability of diagnostic testing for HF in the community. However, the performance of these algorithms and their use along with handheld TTE devices requires the assessment of their performance in unselected ‘real‐world’ populations of patients with suspected (as opposed to confirmed) HF, many of whom may have comorbidities such as chronic obstructive pulmonary disease and obesity that may reduce image quality. Handheld echocardiography has many potential benefits including ease of transport to environments where patients with suspected HF have clinical interactions (e.g. primary care and pharmacies). Handheld devices are less expensive than cart machines.

In this study, we tested the diagnostic accuracy and interchangeability of automated AI reporting of images acquired using handheld TTE with human analysis (both routine clinical care and core laboratory‐based analyses) of cart‐based TTE in a real‐world cohort of patients with suspected HF.

## Methods

### Study design, setting and participants

This was a multicentre, prospective, observational study conducted at five outpatient sites in Glasgow, United Kingdom. Consecutive patients who were attending a clinical HF diagnostic pathway (online supplementary *Figure* *S1*) were prospectively screened for inclusion. The clinical HF diagnostic pathway is between primary care and secondary care and patients are referred by their general practitioner with a clinical suspicion of HF. The exclusion criteria were a prior diagnosis of HF or if the patient was unable or unwilling to provide informed consent. No patients were excluded based on their echocardiogram image quality. The current study was designed before the COVID‐19 pandemic and carried out during the pandemic. This study was performed according to the UK Policy Framework for Health and Social Care Research and the Declaration of Helsinki and was approved by the West of Scotland Research Ethics Committee (20/SW/0182) and the Health Research Authority. All patients provided written informed consent. This study is registered at ClinicalTrials.gov identifier: NCT04724200. N‐terminal pro‐B‐type natriuretic peptide (NT‐proBNP) testing was repeated at the time of the study assessment (Roche CARDIAC proBNP+ assay run on the Roche cobas h232 instrument).

### Echocardiography

Patients who consented had two echocardiograms performed by one sonographer on the same day at the same clinic visit in the following order:

1. A handheld echocardiogram, using a Phillips® Lumify scanner. The scanning protocol for the handheld included two‐dimensional (including color flow Doppler) parasternal long‐axis, apical two‐, three‐, and four‐chamber views.
2. A conventional TTE scan (‘cart‐based’ scan), performed according to current guidelines^4^, ^7^ using a GE® Vivid E95 scanner. All TTEs were performed by accredited sonographers or cardiologists (either European Association of Cardiovascular Imaging or British Society of Echocardiography accredited) (HH, KT, PT, PP, FG).

### Echocardiogram analysis

The following echocardiogram analyses were performed:

1. Handheld echocardiography reported by Us2.ai® (commercially available, CE marked and Food and Drug Administration‐approved software for automated AI analysis, version 1.4.1)
2. Handheld echocardiography reported by two blinded core laboratory sonographers.
3. Cart‐based echocardiogram reported by the clinical sonographer as part of usual care at the time of clinic appointment.
4. Cart‐based echocardiogram reported by a blinded core laboratory sonographer.
5. Cart‐based echocardiogram reported by Us2.ai automated AI analysis

Core laboratory assessors were blinded to all measurement performed by the usual care sonographer. The Us2.ai software allows for fully automated and unsupervised analysis, where the entire echocardiogram (in Digital Imaging and Communications in Medicine format) is analysed, with no need for manual classification of image sequence. The development, training, and validation of the deep‐learning algorithm and workflow are described in detail elsewhere.^5^, ^6^ An example of the automated report is shown in online suppplementary *Figure* *S2*.

The clinical TTE report performed during usual care was obtained using GE® EchoPac (version 2.04) and manually reported in keeping with contemporary guidelines.^7^ Simpson's biplane measurement of LVEF was performed manually (i.e. not using the semi‐automated analysis available in EchoPac). Blinded core laboratory sonographers (RTC/KM/SN/MMYL/PP/JC/PS/JJ) used StudyCast® software. Echocardiograms analysed by humans in the core laboratory were anonymized.

### Outcomes

#### Primary endpoint

The primary endpoint was the diagnostic accuracy of AI‐automated analysis of handheld TTE to identify an LVEF ≤40%. The ‘gold standard’ used for the purposes of comparison with the AI‐automated analysis of handheld TTE was the assessment of LVEF during the human analysis of the cart‐based TTE images as part of usual clinical care. Diagnostic accuracy was defined as: (true positive + true negative)/(true positive + true negative + false positive + false negative). Sensitivity, specificity, negative and positive predictive values and area under the receiver operating characteristic (ROC) curve were also calculated.

#### Secondary endpoints

Secondary endpoints included: (i) the interchangeability (assessed by individual equivalence coefficient [IEC]) of LVEF as measured by AI‐automated handheld TTE and human reporting of cart‐based TTE (both usual care clinical and core laboratory reports); and (ii) the interchangeability of echocardiographic measures of HF with preserved ejection fraction (HFpEF) between AI‐automated and human reporting of cart‐based TTE (both usual care clinical and core lab reports).

#### Exploratory endpoints

Exploratory endpoints included the interchangeability of AI‐automated cart‐based LVEF compared to human reporting of cart‐based LVEF (both usual care clinical and core laboratory reports).

The proportion of all scans where an LVEF was calculated by each echocardiography method was also reported. The agreement between AI‐automated analyses of LVEF (for both handheld and cart‐based images) and human core laboratory reports, and the agreement between AI‐automated assessment of echocardiographic measures of HFpEF and human core laboratory reports (for cart‐based images) were reported.

### Statistical analyses

The primary endpoint of the diagnostic accuracy of AI‐automated analysis of handheld TTE to identify an LVEF ≤40% compared to human clinical assessment of LVEF ≤40% was assessed by diagnostic accuracy, negative and positive predictive value, false positive, false negative, sensitivity, specificity, and area under the ROC curve.

The secondary endpoints comparing the interchangeability of LVEF as measured by AI‐automated analysis and human analyses of cart‐based TTE were assessed by the IEC. The IEC is a measure of the level of disagreement between AI‐automated measurements and human measurements relative to the disagreement among the human measurements. For the purposes of analysis of the IEC in this study, human measurements refer to the results provided by the human‐based usual care clinical and human‐based core laboratory TTE reports (i.e. two human reports). The IEC can be calculated using the following formula: IEC = [*Q*_TR_ − *Q*_RR_]/(*Q*_RR_/2). *Q*_RR_ refers to the mean of the squared differences between two pairs of within‐patient human (reference) measurements, and *Q*_TR_ refers to the mean of the squared differences between within‐patient measurements from the automated analysis and each of two human measurements. An IEC of less than 0 (i.e. a negative value) indicates less variability between AI‐automated analysis and human analysis than the variability between the human analyses (i.e. between the human‐based usual care clinical and core laboratory TTE reports), and a value greater than 0 (i.e. a positive value) indicates greater variability between automated analysis and humans than between the human analyses. As per previous reports, we defined interchangeability between the imaging reporting modalities as an upper bound of the 95% confidence interval (CI) of the IEC of <0.25.^5^ Agreement between AI‐automated (both handheld and cart‐based scans) and human core laboratory analyses of the cart‐based scans was assessed using Pearson correlation coefficients and presented graphically as Bland–Altman plots.

### Image quality

All TTEs (both handheld and cart‐based scans) were assessed for image quality by the core laboratory sonographers. Image quality was categorized into five groups (excellent, good, fair, poor, technically difficult) (online supplementary *Table* *S1*). IEC for both handheld and cart scans were presented in the five categories of image quality.

### Sample size calculation

Clinical audits of the diagnostic pathway have reported a prevalence of LVEF ≤40% between 7–30% over the preceding 10 years. Therefore, assuming that the prevalence would be 10% and the marginal error of this estimate does not exceed 2%, with 95% confidence level, a population size of 864 participants would be adequately powered to accurately predict the risk of LVEF ≤40% in individuals referred from the community for investigation of HF.^8^ From prior studies, sample sizes of 600 participants have been adequately powered to evaluate the accuracy of automatic echocardiogram measurements compared to manual human measurements.^9^, ^10^ Our sample size was expected to provide a population size with enough power to additionally evaluate the accuracy of AI compared to human echocardiogram measurements.

### Study and data management

The Clinical Trials Unit at the Robertson Centre for Biostatistics (University of Glasgow) was responsible for data management and statistical analysis. All analyses were performed using R (version 4.0.0). This study is reported in accordance with the Developmental and Exploratory Clinical Investigations of Decision support systems driven by Artificial Intelligence (DECIDE‐AI) criteria (online supplementary *Table* *S2*).^11^

### Patient involvement

We co‐developed the study with patients with lived experience of HF. This included the design, management, and conduct of the study. A member of a patient‐led charity (Pumping Marvellous) acted as a member of the study steering committee.

## Results

### Baseline characteristics

Between 5 January and 26 August 2021, 1238 patients attended the clinical HF diagnostic pathway, and 867 were enrolled in the study. Baseline characteristics are presented in *Table* *1* and the *Graphical Abstract*. The median age was 77 years and 49% were male. The median body mass index was 31 kg/m^2^. One‐third of patients had a history of atrial fibrillation and 14% had a known diagnosis of chronic obstructive pulmonary disease. Median NT‐proBNP was 613 (288–1159) pg/ml. A diagnosis of HF with reduced ejection fraction (HFrEF) was made in 44 patients (5.6%) (on the human‐reported clinical echocardiogram 41 had an LVEF ≤40% by Simpson's biplane and three had an LVEF ≤40% based on visual assessment of LVEF where a Simpson's biplane was not possible).

**Table 1:** Baseline characteristics

Characteristic	
*n*	867
Age, years	77 (69, 83)
Male sex, *n* (%)	428 (49)
White race, *n* (%)	823 (98)
BMI, kg/m^2^	31 (27, 36)
SBP, mmHg	148 (131, 165)
Heart rate, bpm	73 (64, 85)
Past medical history, *n* (%)	
Hypertension	501 (59)
Type 2 diabetes	169 (20)
Myocardial infarction	106 (13)
Chronic kidney disease	167 (20)
Atrial fibrillation	275 (33)
Chronic obstructive pulmonary disease	117 (14)
Physical examination, *n* (%)	
Elevated JVP (>4 cm)	39 (6)
Pulmonary crepitations	83 (17)
Peripheral oedema	571 (69)
Baseline blood tests	
NT‐proBNP, pg/ml	613 (288, 1159)
eGFR, ml/min/1.73 m^2^	74 (58, 88)
HbA1c, mmol/mol	41 (38, 45)
HF with reduced ejection fraction, *n* (%)	44 (6)
Clinical cart‐based echocardiogram	
LVEF, %	62 (54, 66)
LVIDd, cm	4.6 (4.2, 5.0)
HFpEF parameters	
E/e'	10.2 (8.2, 12.9)
LAVI, ml/m^2^	37 (29, 49)
LVMI, g/m^2^	94 (79, 114)
TR velocity, m/s	2.6 (2.3, 2.8)

Values expressed as *n* (%) or median (quartile 1, quartile 3).

BMI, body mass index; eGFR, estimated glomerular filtration rate; HbA1c, glycated haemoglobin; HF, heart failure; HFpEF, heart failure with preserved ejection fraction; JVP, jugular venous pressure; LAVI, left atrial volume index; LVEF, left ventricular ejection fraction; LVIDd, left ventricular internal diameter in diastole; LVMI, left ventricular mass index; NT‐proBNP, N‐terminal pro‐B‐type natriuretic peptide; SBP, systolic blood pressure; TR, tricuspid regurgitation.

### Left ventricular ejection fraction reporting by artificial intelligence‐automated and humans

#### Handheld transthoracic echocardiography

**Figure 1:**
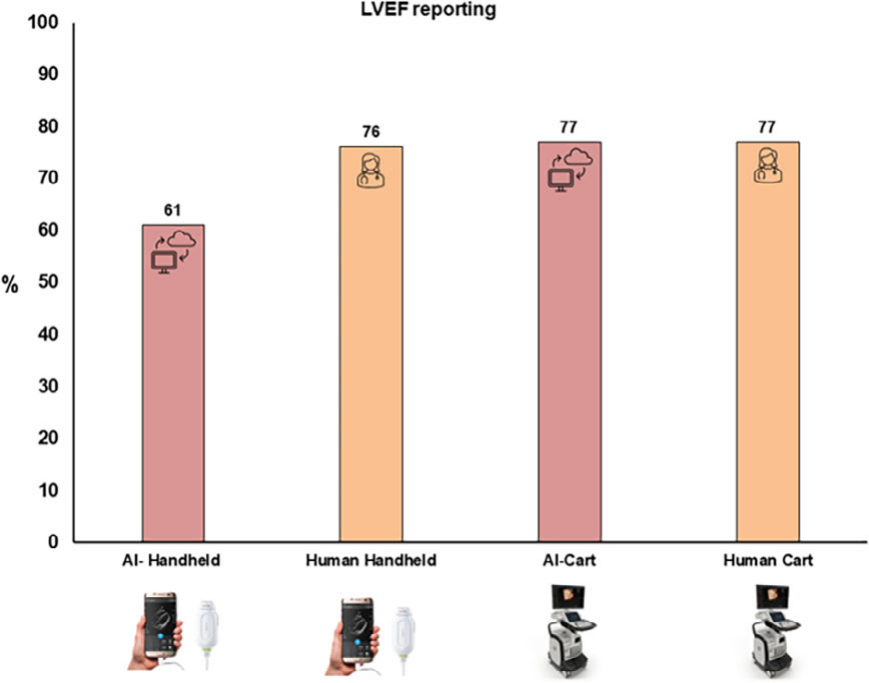
Percentage of left ventricular ejection fraction (LVEF) reported by artificial intelligence (AI) and humans with handheld and cart echocardiograms.

On the handheld device, an LVEF was reported in 61% of scans when analysed by the AI‐automated software. This is compared with an LVEF reported in 83% and 69% of the two human core laboratory readers, respectively, with an average human core laboratory reporting rate of 76% (*Figure* *1*).

#### Cart‐based transthoracic echocardiography

On the cart‐based TTE images, AI‐automated reporting resulted in a similar number of LVEF reports (77%) as the human‐based usual care clinical report (72%) and the human core laboratory report (81%) (the average of the usual care and core laboratory reports was 77%). The median LVEF across each of the different methods of assessment is shown in online supplementary *Figure* *S4*.

### Outcomes

#### Primary endpoint

The primary endpoint of diagnostic accuracy of AI‐automated analysis of handheld TTE to identify an LVEF ≤40%, compared to the report of the clinical human on a cart echocardiogram, was 0.93 (95% CI 0.90, 0.95). The sensitivity was 0.61 (95% CI 0.42, 0.78), specificity 0.95 (95% CI 0.93, 0.97), positive predictive value 0.50 (95% CI 0.33, 0.67), negative predictive value 0.97 (0.95, 0.99), with an area under the ROC curve of 0.96 (95% CI 0.94–0.98). These results are also presented as a confusion matrix in online supplementary *Table* *S3*.

#### Secondary endpoints

The LVEF reported by the AI‐automated analysis of the handheld TTE was interchangeable with the LVEF reported by the analysis of cart‐based TTE by two human sonographers (usual care clinical and core laboratory reports) (IEC −0.40, 95% CI −0.60, −0.16).

The IEC for additional chamber dimensions and echocardiographic measures of HFpEF are shown in *Table* *2*. For cart‐based echocardiograms, Doppler measurements were interchangeable between AI‐automated reports and two human reports of cart echocardiograms (usual care clinical and core laboratory reports). Although the point estimate for IEC for left atrial volume was under 0.25, 95% CIs were wide, therefore it did not meet our pre‐defined definition of interchangeability based on the upper bound of the 95% CI of IEC.

**Table 2:** Individual equivalence coefficient values

Measurement	Automated handheld analysis			Automated cart analysis		
	Scans with 3 assessments (*n*)^a^	IEC (95% CI)	Interchangeable	Scans with 3 assessments (*n*)^a^	IEC (95% CI)	Interchangeable
Ventricular volumes and dimensions						
LVEF	410	−0.40 (−0.60, −0.16)	Yes	521	−0.39 (−0.60, −0.12)	Yes
LVEDV	410	0.61 (0.16, 1.21)	No	522	0.04 (−0.30, 0.49)	No
LVESV	411	0.18 (−0.18, 0.63)	No	524	−0.12 (−0.48, 0.35)	Yes
LVIDd	640	1.73 (1.19, 2.41)	No	712	−0.01 (−0.25, 0.29)	No
LVIDs	553	0.04 (−0.28, 0.45)	No	620	−0.32 (−0.51, −0.10)	Yes
IVSDd	589	−0.06 (−0.30, 0.25)	Yes	672	0.24 (−0.03, 0.56)	No
LVPWd	566	−0.70 (−0.84, −0.52)	Yes	668	−0.44 (−0.60, −0.25)	Yes
RAA	325	0.39 (0.05, 0.82)	No	385	−0.14 (−0.39, 0.20)	Yes
RVIDd	435	0.00 (−0.29, 0.35)	No	551	−0.26 (−0.49, −0.02)	Yes
TAPSE		–		316	−0.12 (−0.51, 0.41)	No
HFpEF echocardiographic markers						
LA volume	244	2.23 (1.31, 3.54)	No	322	0.19 (−0.12, 0.57)	No
LV mass	541	0.61 (0.28, 1.03)	No	638	0.91 (0.53, 1.37)	No
MV E	–	–	–	410	−0.86 (−0.98, −0.69)	Yes
MV A	–	–	–	262	−0.81 (−1.03, −0.52)	Yes
MV E/A	–	–	–	252	−0.95 (−1.12, −0.45)	Yes
MV Dec T	–	–	–	324	−0.39 (−0.68, −0.01)	Yes
E/e' average	–	–	–	319	−0.82 (−0.98, −0.58)	Yes
TV peak velocity	–	–	–	287	−0.66 (−0.83, −0.41)	Yes

CI, confidence interval; Dec T, deceleration time; HFpEF, heart failure with preserved ejection fraction; IEC, individual equivalence coefficient; IVSDd, interventricular septal diameter in diastole; LA, left atrial; LV, left ventricular; LVEDV, left ventricular end‐diastolic volume; LVEF, left ventricular ejection fraction; LVESV, left ventricular end‐systolic volume; LVIDd, left ventricular internal diameter in diastole; LVIDs, left ventricular internal diameter in systole; LVPWd, left ventricular posterior wall in diastole; MV, mitral valve; RAA, right atrial area; RVIDd, right ventricular internal diameter in diastole; TAPSE, tricuspid annular plane systolic excursion; TV, tricuspid valve.

^a^ Two human cart analyses and one automated AI analysis.

#### Exploratory analyses

Artificial intelligence‐automated analysis of the LVEF from cart‐based TTE images was interchangeable with the LVEF reported by two human sonographers on cart‐based TTE images (IEC −0.39, 95% CI −0.60, −0.12).

In a *post hoc* analysis, we compared the IEC of AI fully automated analysis of handheld scans to two human core laboratory‐blinded analyses of the same handheld scans (rather than comparing to the cart scan). The IEC showed a consistent result regarding LVEF assessment, with an IEC of −0.34 (95% CI −0.53, −0.11).

**Figure 2:**
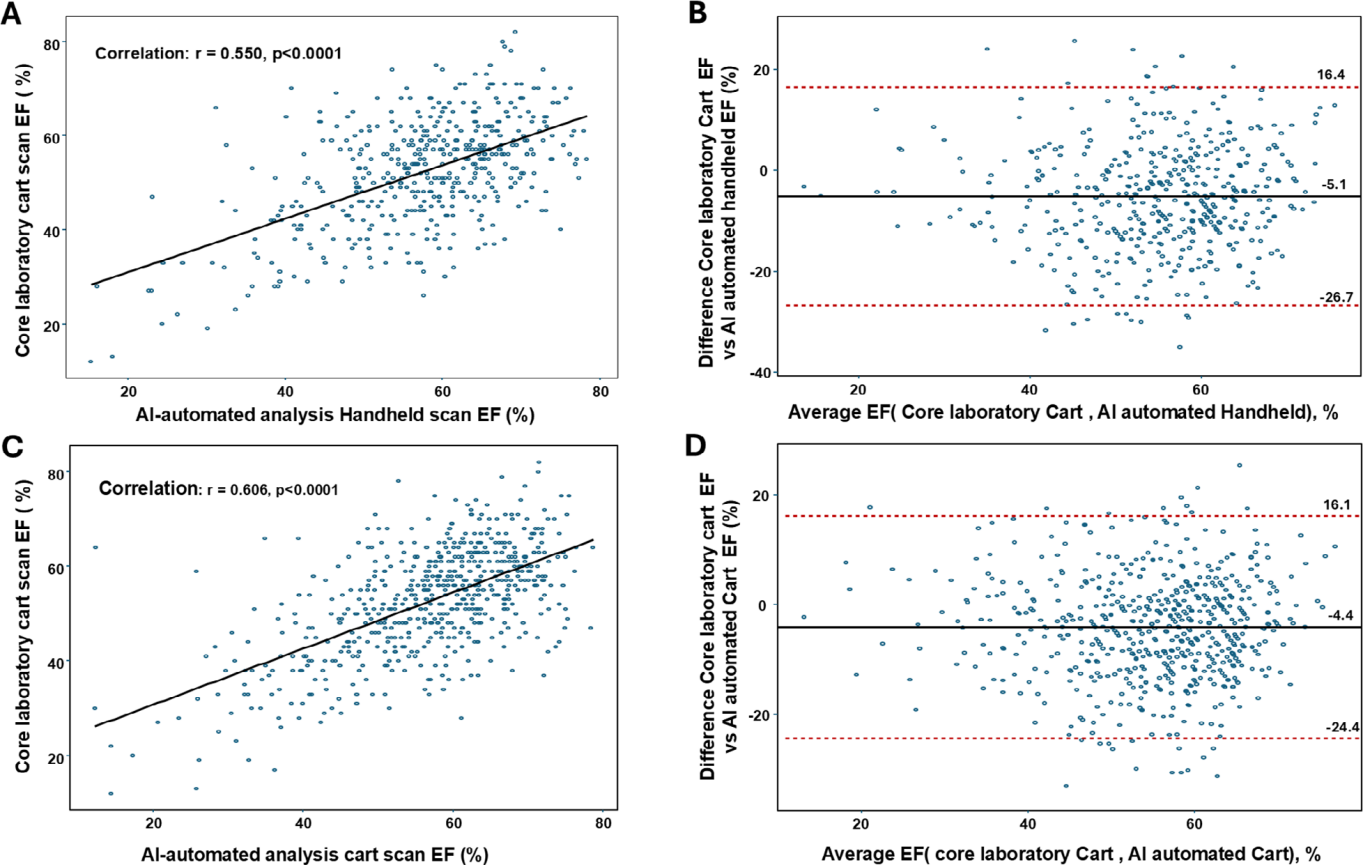
Correlation and Bland–Altman plots of artificial intelligence (AI)‐automated measurement (handheld and cart) with cart core laboratory ejection fraction (EF). (*A*) Scatter plot of AI‐automated handheld EF and core laboratory cart EF. (*B*) Bland–Altman plot comparing AI‐automated handheld EF and core laboratory cart EF. (*C*) Scatter plot of AI‐automated cart EF and core laboratory cart EF. (*D*) Bland–Altman plot comparing AI‐automated cart EF and core laboratory cart EF.

In a second *post hoc* analysis, we compared the agreement between AI‐automated analysis of LVEF on both handheld and cart scans with the human core laboratory analysis of the cart scans (*Figure* *2*). We also compared the agreement between core laboratory cart and other automated echocardiographic measures including those used in the assessment of HFpEF (on cart‐based scans only, online supplementary *Figures* *S5* *–* *S21*). The results of these analyses are presented in the online *Supporting Information*. There was a strong correlation between AI‐automated and human core laboratory LVEF (Pearson *r* = 0.61, *p* < 0.0001) (*Figure* *2*), and between AI‐automated handheld and human core laboratory LVEF (*r* = 0.55, *p* < 0.0001). The lowest correlation coefficient was seen with mitral valve deceleration time (*r* = 0.34, *p* < 0.001), and the highest with peak mitral valve E velocity (*r* = 0.94, *p* < 0.0001). The mean difference between AI‐automated LVEF and human core laboratory analyses for handheld scans was 5.2% (95% CI 4.2, 6.2; *p* < 0.0001). The mean difference between AI‐automated LVEF and human core laboratory analyses for cart scans was 4.2% (95% CI 3.4, 5.0); (*p* < 0.0001). Both of these mean differences were smaller than the difference between the human clinical assessment and human core laboratory measurements of cart scans (6.5%, 95% CI 5.7, 7.3; *p* < 0.0001).

In a third *post hoc* analysis, we compared the diagnostic accuracy of AI‐automated analysis of handheld TTE to identify an LVEF <50%, compared to the report of the clinical human on a cart echocardiogram. The results (shown in online supplementary *Tables* *S4* and *S5*) were consistent with the primary pre‐specified analysis.

### Image quality

**Figure 3:**
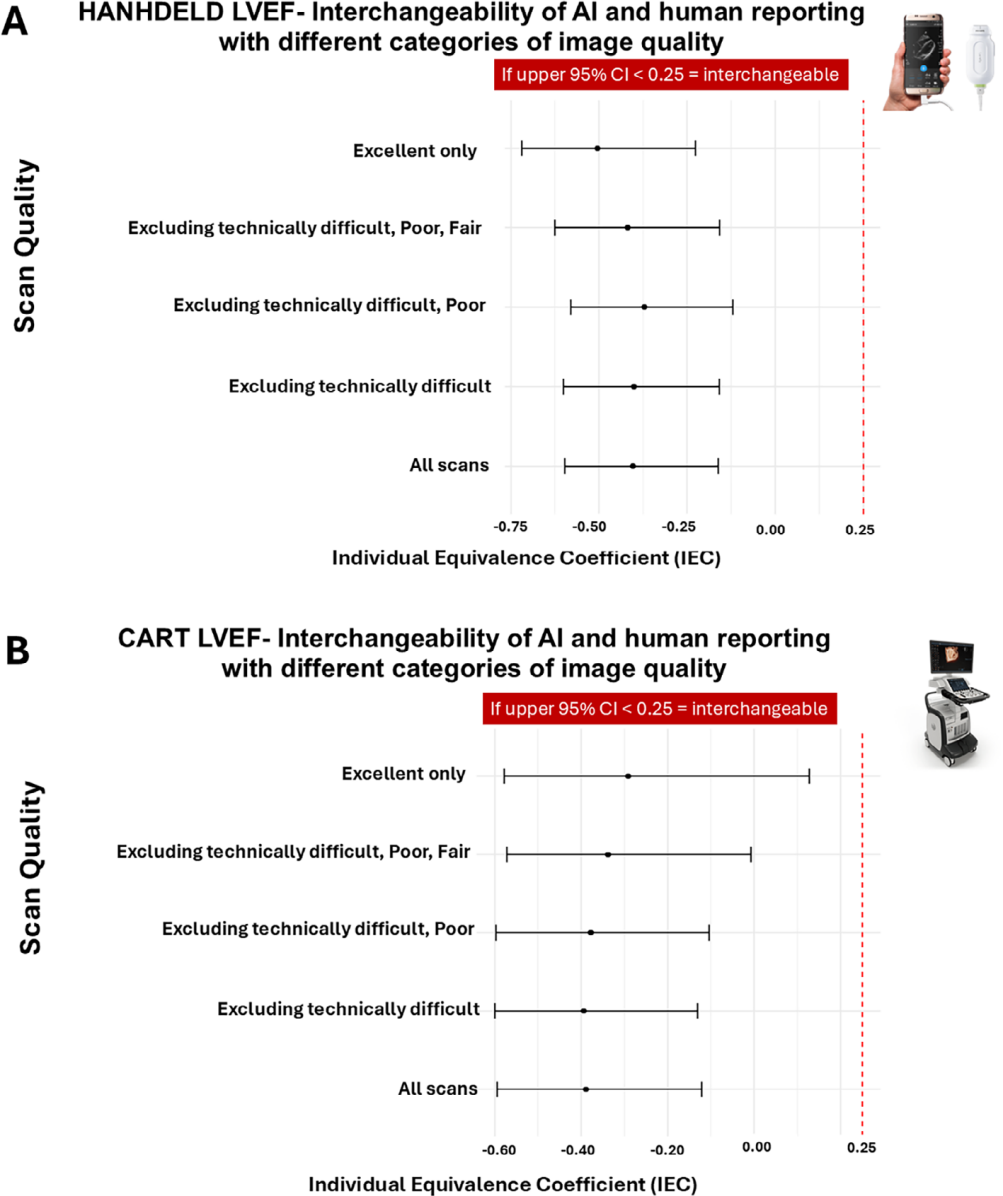
Interchangeability of of artificial intelligence (AI) and human reporting of left ventricular ejection fraction (LVEF) on handheld (*A*) and cart (*B*) echocardiograms according to image quality. CI, confidence interval.

The interchangeability of LVEF reporting with AI‐automated reporting and two humans (usual care clinical and core laboratory reports) was seen regardless of image quality on both handheld and cart scans (*Figure* *3*). Image quality was categorized as substantially worse on handheld than on cart echocardiograms (online supplementary *Figure* *S3*).

## Discussion

We conducted a large prospective, real‐world assessment of AI‐automated reporting of handheld echocardiograms compared to human reporting of cart‐based TTE. We have shown that the AI‐automated analysis of images acquired with a handheld scanner had good diagnostic performance for detecting an LVEF ≤40% (when compared to humans in daily clinical practice). We have also shown that AI‐automated LVEF was interchangeable with LVEF reported by humans on a cart‐based TTE in a clinical setting. AI‐automated LVEF reporting on a cart is also interchangeable with human reporting of cart‐based TTE. On cart scans, AI‐automated reporting met criteria for interchangeability with human reporting of most, but not all, echocardiographic parameters suggested in guidelines to be used in the diagnosis of HFpEF.^1^ The interchangeability of LVEF reporting between AI‐automated reporting and humans was seen regardless of image quality on both handheld and cart scans.

Our study was large and prospective, with 867 near‐consecutive patients evaluated as part of a routine clinical diagnostic pathway. This is in contrast to most previous studies assessing the utility of semi‐automated and fully automated AI algorithms for analysis of echocardiograms which were mostly retrospective. In contrast to previous validation cohorts, the population included in the present study had substantial comorbidity (e.g. >50% were obese). Many previous studies used core laboratory echocardiography scans from selected populations (usually from clinical trial or research study populations). We did not exclude patients with poor acoustic windows, although we were able to only compare scans where an ejection fraction was produced by both human and automated analyses. Although prior studies have been very useful for the development and early validation of AI‐automated algorithms, questions remained regarding their utility in ‘real‐world’ patients assessed in everyday clinical practice. One of the key strengths of our study was the focus on ‘real‐world’, consecutive patients referred by their primary care physician for a clinically indicated diagnostic TTE.

Our primary endpoint focused on the ability of AI‐automated reporting of handheld scans to diagnose those with an LVEF ≤40% (i.e. the echocardiographic biomarker of the syndrome of HFrEF). We compared its performance to sonographers' reporting on a cart‐based TTE in a routine clinical setting. This was deemed to be the most clinically meaningful standard of comparison for the combined AI‐handheld TTE approach. A key goal of HF diagnostic pathways is to identify very high‐risk patients where there is a major treatment opportunity. In patients with HFrEF, comprehensive medical therapy can reduce mortality by around 50% and hospitalizations for HF by 60–70%.^12^ The diagnostic accuracy of AI‐automated reporting of LVEF ≤ 40% was high. The negative predictive value was 0.97. The positive predictive value (0.50) suggests that those who are thought to have LVEF ≤40% by AI handheld assessment require a confirmatory cart‐based scan, although this lower positive predictive value will be influenced by the relatively low prevalence of HFrEF in the cohort. The high negative predictive value is of importance, excluding patients who do not have LVEF ≤40%. By using AI‐automated handheld TTE, HFrEF could be excluded efficiently and those with potential HFrEF identified. The use of a handheld TTE potentially allows the diagnosis of HFrEF to be moved from secondary care into primary or community care.

We also found that LVEF reported by AI on handheld devices was interchangeable with LVEF reported by humans reporting cart images in a clinical environment, although this was only where both AI‐automated analysis and two human analyses were possible. This finding has major clinical implications. For example, many echocardiograms are requested to investigate recovery of myocardial function after HF therapy has been instituted. Among other clinical implications, the degree of myocardial recovery determines eligibility for cardiac resynchronization therapy or implantable cardioverter‐defibrillators. A focused, rapid, accurate assessment of LVEF by AI reporting on a handheld device in this setting is potentially of great clinical value, negating the need for a time‐consuming cart‐based scan and human reporting. The value of AI reporting of LVEF on handheld machines has major potential, especially in regions of the world where there is a lack of cart machines and a dearth of expert reporting sonographers. The impact on many aspects of HF diagnosis and management could be profound.

Artificial intelligence‐automated reporting of LVEF on cart images was also interchangeable with human reporting of cart images. Obtaining an AI‐automated LVEF report, which is equivalent to a human sonographer, would result in major time‐saving and efficiencies, through workflow optimization. Importantly AI reporting of cart scans resulted in similar numbers of LVEF reports as cart scans reported by human clinical sonographers. Workflow acceleration has recently been demonstrated using this approach in clinical echo labs where AI‐automated echo reporting was shown to reduce echo analysis time by 70% without compromising accuracy.^13^ Such efficiency benefits are similar to other areas of diagnostic imaging where AI‐automated algorithms are being deployed in clinical care.^14^ This workflow acceleration could increase the number of scans performed per sonographer/scanner and therefore increase capacity in the systems which are often working beyond capacity. By increasing the throughput of an echocardiogram department, the time to diagnostic scan for HF would likely reduce, and this in turn has the potential to allow patients to be started on HFrEF disease‐modifying therapies sooner. As AI‐automated analysis models and handheld echocardiographic equipment continue to improve, it is anticipated that there are likely to be further gains in diagnostic accuracy and in the proportion of patients where automated analysis is possible.

### Limitations

A key strength of our study is that every TTE, both handheld and cart‐based, was performed by accredited sonographers/cardiologists. All of the blinded analyses were also performed by expert/accredited sonographers. This could limit the potential generalizability, with our results only applicable to scanning performed by accredited sonographers and cardiologists. It is probable, although we did not formally test this, that a novice acquisition of images would result in a lower yield of LVEF assessment. A potential source of bias in our study was the timing of the handheld scan in relation to the cart scan. In our study the same sonographer performed both handheld and cart scans with the handheld scan being performed first in each case. It may be that the sonographer was able to acquire better images on the second scan. It was not possible to calculate an LVEF in every patient on every scan. The assessment of the primary endpoint was only possible in 445 of 867 (51%) participants. This was anticipated and expected, with estimates of 10–15% of patients having a TTE requiring contrast agents to allow accurate endocardial definition and LVEF calculation.^15^ To obtain an LVEF in every patient, ultrasound enhancing contrast agents would be required for the patients with limited acoustic windowing. We elected not to administer contrast agents for those with poor acoustic windows as the AI‐automated algorithm had not been trained on datasets utilizing contrast echocardiography. Although rates of LVEF reporting on the handheld scans were lower than cart‐based scans, AI reporting could still represent a potential major step forward in a triage setting, particularly when used by a trained sonographer. Very few non‐White patients were enrolled and the study was performed in one country. Our results require validation in other populations.

## Conclusions

Artificial intelligence‐automated reporting of LVEF ≤40% on handheld devices has good diagnostic accuracy compared to humans reporting cart LVEF. AI reporting of LVEF on handheld devices and cart scanners is interchangeable with human cart reporting. Incorporating AI‐automated reporting of TTE has the potential to accelerate exclusion, identification and treatment of HFrEF.

## Supporting information


**Appendix S1.** Supporting Information.
