# Supplementary material for: Artificial intelligence fully automated analysis of handheld echocardiography in real‐world patients with suspected heart failure
Source: Eur J Heart Fail. 2025 Jul 24;27(12):3401–10. doi: 10.1002/ejhf.3783 (PMC12803686; doi:10.1002/ejhf.3783)

**Supplementary appendix**

# **Supplementary appendix table of contents**

1. Supplementary table S1: Echocardiography image quality assessment criteria yield of handheld echocardiogram automated analysis
2. Supplementary table S2: DECIDE-AI reporting guideline checklist
3. Supplementary table S3- confusion matrix for diagnosis of LVEF ≤40% by AI automated handheld compared to Human Cart LVEF ≤40%
4. Supplementary table s4- confusion matrix for diagnosis of LVEF <50% by AI automated handheld compared to Human Cart LVEF <50%
5. Supplementary table s5 Diagnostic Performance of AI automated handheld LVEF compared to LVEF measurements Human Cart LVEF (HFrEF/HFmrEF diagnosis)
6. Supplementary table s6- missingness baseline table
7. Supplementary figure S1- HFDP flow chart
8. Supplementary figure S2- example automated report
9. Supplementary figure S3: Image quality assessment
10. Supplementary figure S4 : LVEF assessed by different modalities
11. Supplementary figure S5-21: correlations between core laboratory and AI-automated measured LVEDV, LVIDd, LVIDs, RVIDd, TAPSE, LA volume, LV mass, MV E, MV A, MV E/A, MV dec time, E/E’, TR Vmax

**Supplementary table 1: Assessment of echocardiogram image quality**

| Assessment of image quality | Criteria | Simpson’s biplane possible to calculate |
| --- | --- | --- |
| 1. ‘Technically difficult’ | - Poor image quality. - Inadequate four and two chamber apical views for modified Simpson’s biplane, major issues with apical views of either 2 or 4 chamber (off axis, foreshortened, myocardium outside image, missing 2 or 4 chamber ) - Inadequate endocardial / blood pool definition | - no |
| 1. ‘poor’ | - Poor but diagnostic image quality. - Adequate four and two chamber apical views for modified Simpson’s biplane , or some deficiencies in image acquisition but overall possible to perform ejection fraction (off axis, foreshortened, myocardium outside image ) - Lower quality endocardial / blood pool definition, but possible to mark endocardial border | - yes |
| 1. ‘Fair’ | - Adequate image quality. - Good four and two chamber apical views for modified Simpson’s biplane , only minimal issues with apical views of either 2 or 4 chamber (off axis, foreshortened, myocardium outside image, missing 2 or 4 chamber ) - Adequate quality endocardial / blood pool definition | - yes |
| 1. ‘Good’ | - Adequate image quality. - Good four and two chamber apical views for modified Simpson’s biplane , only minimal issues with apical views of either 2 or 4 chamber (off axis, foreshortened, myocardium outside image, missing 2 or 4 chamber ) - Adequate quality endocardial / blood pool definition | - yes |
| 1. ‘Excellent’ | - Excellent image quality. - Excellent four and two chamber apical views for modified Simpson’s biplane - Excellent quality endocardial / blood pool definition | - yes |

**Supplementary table S2: DECIDE-AI reporting guideline checklist**

| Item No | Theme | Recommendation | Reported on page |
| --- | --- | --- | --- |
| **1-17** | **AI specific reporting items** | |  |
| I-X | Generic reporting items | |  |
| **Title and abstract** | | |  |
| **1** | **Title** | **Identify the study as early clinical evaluation of a decision support system based on AI or machine learning, specifying the problem addressed** | **1** |
| I | Abstract | Provide a structured summary of the study. Consider including: intended use of the AI system, type of underlying algorithm, study setting, number of patients and users included, primary and secondary outcomes, key safety endpoints, human factors evaluated, main results, conclusions | **2** |
| **Introduction** | | |  |
| **2** | **Intended use** | **a) Describe the targeted medical condition(s) and problem(s), including the current standard practice, and the intended patient population(s)** | **2** |
|  |  | **b) Describe the intended users of the AI system, its planned integration in the care pathway, and the potential impact, including patient outcomes, it is intended to have** | **2** |
| II | Objectives | State the study objectives | 2 |
| **Methods** | | |  |
| III | Research governance | Provide a reference to any study protocol, study registration number, and ethics approval | 3 |
| **3** | **Participants** | **a) Describe how patients were recruited, stating the inclusion and exclusion criteria at both patient and data level, and how the number of recruited patients was decided** | 5 |
|  |  | **b) Describe how users were recruited, stating the inclusion and exclusion criteria, and how the intended number of recruited users was decided** | 5 |
|  |  | **c) Describe steps taken to familiarise the users with the AI system, including any training received prior to the study** | - |
| **4** | **Al system** | **a) Briefly describe the AI system, specifying its version and type of underlying algorithm used. Describe, or provide a direct reference to, the characteristics of the patient population on which the algorithm was trained and its performance in preclinical development/validation studies** | 6 |
|  |  | **b) Identify the data used as inputs. Describe how the data were acquired, the process needed to enter the input data, the pre-processing applied, and how missing/low-quality data were handled** | 6 |
|  |  | **c) Describe the AI system outputs and how they were presented to the users (an image may be useful)** | - |
| **5** | **Implementation** | **a) Describe the settings in which the AI system was evaluated** | 6 |
|  |  | **b) Describe the clinical workflow/care pathway in which the AI system was evaluated, the timing of its use, and how the final supported decision was reached and by whom** | 6 |
| IV | Outcomes | Specify the primary and secondary outcomes measured | 6,7 |
| **6** | **Safety and errors** | **a) Provide a description of how significant errors/malfunctions were defined and identified** | **7,8** |
|  |  | **b) Describe how any risks to patient safety or instances of harm were identified, analysed, and minimised** | **-** |
| **7** | **Human factors** | **Describe the human factors tools, methods or frameworks used, the use cases considered, and the users involved** | **-** |
| V | Analysis | Describe the statistical methods by which the primary and secondary outcomes were analysed, as well as any prespecified additional analyses, including subgroup analyses and their rationale | 8 |
| **8** | **Ethics** | **Describe whether specific methodologies were utilised to fulfil an ethics-related goal (such as algorithmic fairness) and their rationale** | **-** |
| VI | Patient involvement | State how patients were involved in any aspect of: the development of the research question, the study design, and the conduct of the study | 9 |
| **Results** | | |  |
| **9** | **Participants** | **a) Describe the baseline characteristics of the patients included in the study, and report on input data missingness** | **10** |
|  |  | **b) Describe the baseline characteristics of the users included in the study** | **10** |
| **10** | **Implementation** | **a) Report on the user exposure to the AI system, on the number of instances the AI system was used, and on the users’ adherence to the intended implementation** | **-** |
|  |  | **b) Report any significant changes to the clinical workflow or care pathway caused by the AI system** | **-** |
| VII | Main results | Report on the prespecified outcomes, including outcomes for any comparison group if applicable | 10 , 11 |
| VIII | Subgroups analysis | Report on the differences in the main outcomes according to the prespecified subgroups | 11 |
| **11** | **Modifications** | **Report any changes made to the AI system or its hardware platform during the study. Report the timing of these modifications, the rationale for each, and any changes in outcomes observed after each of them** | **-** |
| **12** | **Human-computer agreement** | **Report on the user agreement with the AI system. Describe any instances of and reasons for user variation from the AI system’s recommendations and, if applicable, users changing their mind based on the AI system’s recommendations** | **11** |
| **13** | **Safety and errors** | **a) List any significant errors/malfunctions related to: AI system recommendations, supporting software/hardware, or users. Include details of: (i) rate of occurrence, (ii) apparent causes, (iii) whether they could be corrected, and (iv) any significant potential impacts on patient care** | **-** |
|  |  | **b) Report on any risks to patient safety or observed instances of harm (including indirect harm) identified during the study** | **11** |
| **14** | **Human factors** | **a) Report on the usability evaluation, according to recognised standards or frameworks** | **-** |
|  |  | **b) Report on the user learning curves evaluation** | **-** |
| **Discussion** | | |  |
| **15** | **Support for intended use** | **Discuss whether the results obtained support the intended use of the AI system in clinical settings** | **13** |
| **16** | **Safety and errors** | **Discuss what the results indicate about the safety profile of the AI system. Discuss any observed errors/malfunctions and instances of harm, their implications for patient care, and whether/how they can be mitigated** | **13** |
| IX | Strengths and limitations | Discuss the strengths and limitations of the study | 14 |
| **Statements** | | |  |
| **17** | **Data availability** | **Disclose if and how data and relevant code are available** | **18** |
| X | Conflicts of interest | Disclose any relevant conflicts of interest, including the source of funding for the study, the role of funders, any other roles played by commercial companies, and personal conflicts of interest for each author | 17 |

AI=artificial intelligence. AI specific items are numbered in Arab numerals, generic items in Roman numerals.

## **Supplementary table s3- confusion matrix for diagnosis of LVEF ≤40% by AI automated handheld compared to Human Cart LVEF ≤40%**

|  |  | **Human Cart LVEF ≤40%** | |
| --- | --- | --- | --- |
|  |  | **Yes** | **No** |
| **AI automated handheld**  **LVEF ≤40%** | **Yes** | **19** | **19** |
|  | **No** | **12** | **395** |

## **Supplementary table s4- confusion matrix for diagnosis of LVEF <50% by AI automated handheld compared to Human Cart LVEF <50%**

|  |  | **Human Cart LVEF <50%** | |
| --- | --- | --- | --- |
|  |  | **Yes** | **No** |
| **AI automated handheld**  **LVEF <50%** | **Yes** | **53** | **43** |
|  | **No** | **25** | **324** |

**Supplementary table s5- Diagnostic Performance of AI automated handheld LVEF <50% compared to LVEF measurements Human Cart LVEF (HFrEF/HFmrEF diagnosis)**

| A LVEF < 50% is classed as a diagnosis of HFrEF/HFmrEF. Confidence intervals are calculated by exact binomial tests (Clopper-Pearson method). | |
| --- | --- |
| Performance Measure | Human Cart LVEF |
| Sensitivity (95% CI) | 0.679 (0.564, 0.781) |
| Specificity (95% CI) | 0.883 (0.845, 0.914) |
| Accuracy (95% CI) | 0.847 (0.810, 0.879) |
| Positive Predictive Value (95% CI) | 0.552 (0.447, 0.654) |
| Negative Predictive Value (95% CI) | 0.928 (0.896, 0.953) |

## **Supplementary table s6- missingness baseline table**

| Characteristic | n (missing) |  |
| --- | --- | --- |
| n |  | 867 |
| Age – years | 867 (0) | 77 (69, 83) |
| Male sex – no. (%) | 867 (0) | 428 (49) |
| White Race– no. (%) | 844 (23) | 823 (98) |
| BMI (kg/m^2^) | 852 (15) | 31 (27, 36) |
| SBP (mmHg) | 849 (18) | 148 (131, 165) |
| Heart rate (bpm) | 854 (13) | 73 (64, 85) |
| Past medical history |  |  |
| Hypertension (n,%) | 844 (23) | 501 (59) |
| Type 2 diabetes (n,%) | 844 (23) | 169 (20) |
| Myocardial infarction (n,%) | 844 (23) | 106 (13) |
| Chronic kidney disease (n,%) | 844 (23) | 167 (20) |
| Atrial fibrillation (n,%) | 844 (23) | 275 (33) |
| Chronic obstructive pulmonary disease (n,%) | 844 (23) | 117 (14) |
| Physical Examination |  |  |
| Elevated JVP (>4cm) (n,%) | 610 (257) | 39 (6) |
| Pulmonary crepitations (n,%) | 491 (376) | 83 (17) |
| Peripheral oedema (n,%) | 826 (41) | 571 (69) |
| Baseline Blood tests |  |  |
| NT-proBNP (pg/mL) | 842 (25) | 613 (288, 1159) |
| eGFR (mL/min/1.73m^2^) | 825 (42) | 74 (58, 88) |
| HbA1c (mmol/mol) | 805 (62) | 41 (38, 45) |
| HF with reduced ejection fraction (n,%) |  | 41 (6) |
| Clinical cart-based echocardiogram |  |  |
| LVEF, % | 627 (240) | 62^$^ (54, 66) |
| LVIDD, cm | 845 (22) | 4.6 (4.2, 5.0) |
| HFpEF parameters |  |  |
| E/e’ | 697 (170) | 9.8 (8.5, 11.6) |
| LAVI, ml/m^2^ | 687 (180) | 25 (17, 36) |
| LVMI, g/m^2^ | 789 (78) | 114 (95, 141) |
| TR velocity , m/s | 563 (295) | 2.6 (2.3, 2.8) |
| Values expressed as n(%) or median (Quartile 1, Quartile 3).  ^$^ Ejection fraction from clinical transthoracic echocardiogram  eGFR= estimated glomerular filtration rate; HbA1c= haemoglobin A1c; HFrEF= heart failure with reduced ejection fraction; HFpEF = heart failure with preserved ejection fraction; LAVI = left atrial volume index; LVEF= left ventricular ejection fraction; LVIDD= left ventricular internal diameter in diastole; LVMI= left ventricular mass index;; SBP= systolic blood pressure; TR= tricuspid regurgitation. | | |

**Supplementary Figure S1- Heart failure diagnostic pathway flow chart**

**Supplementary Figure S2- Example of a US2ai report**

**
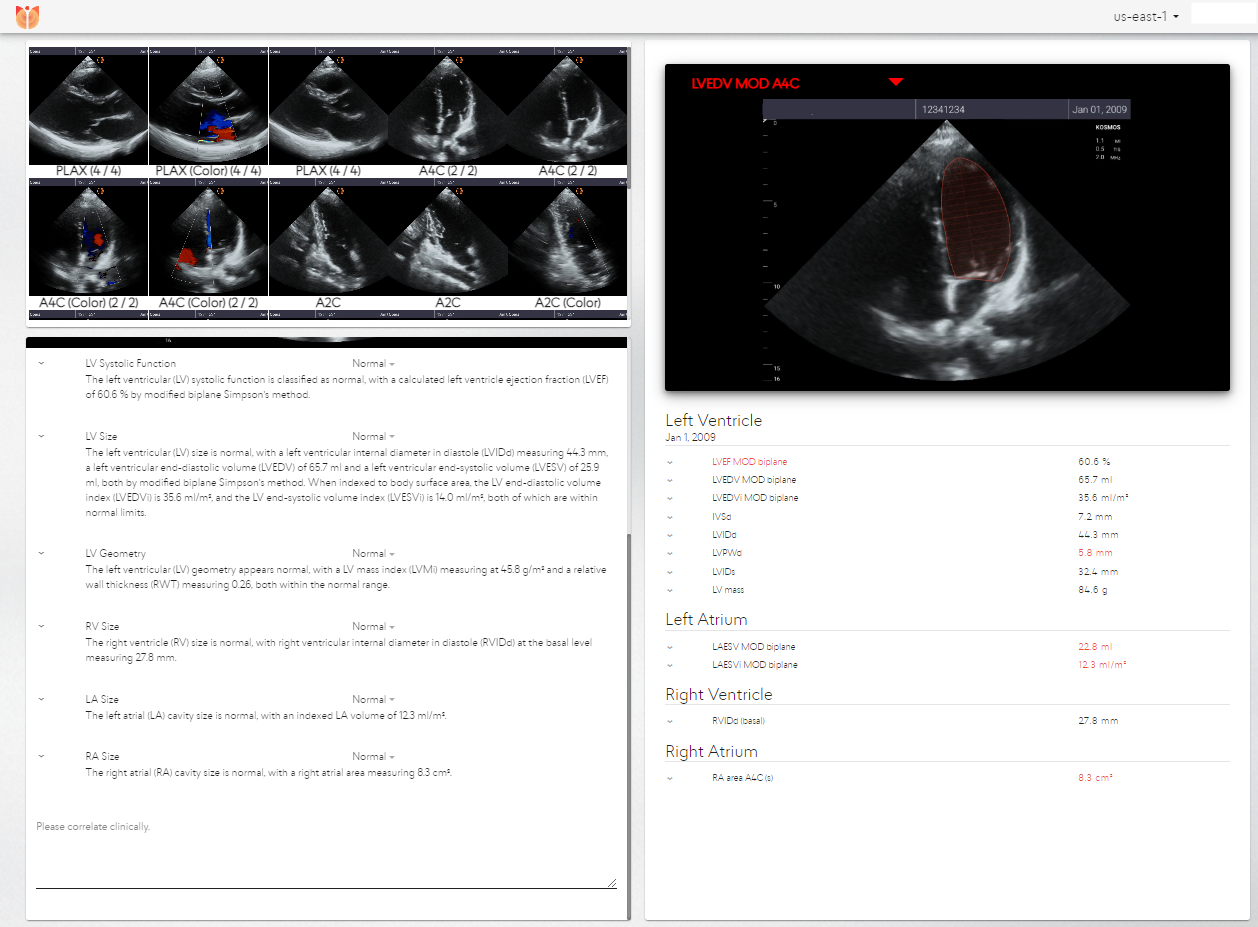
**

**Supplementary Figure S3**: Image quality assessment


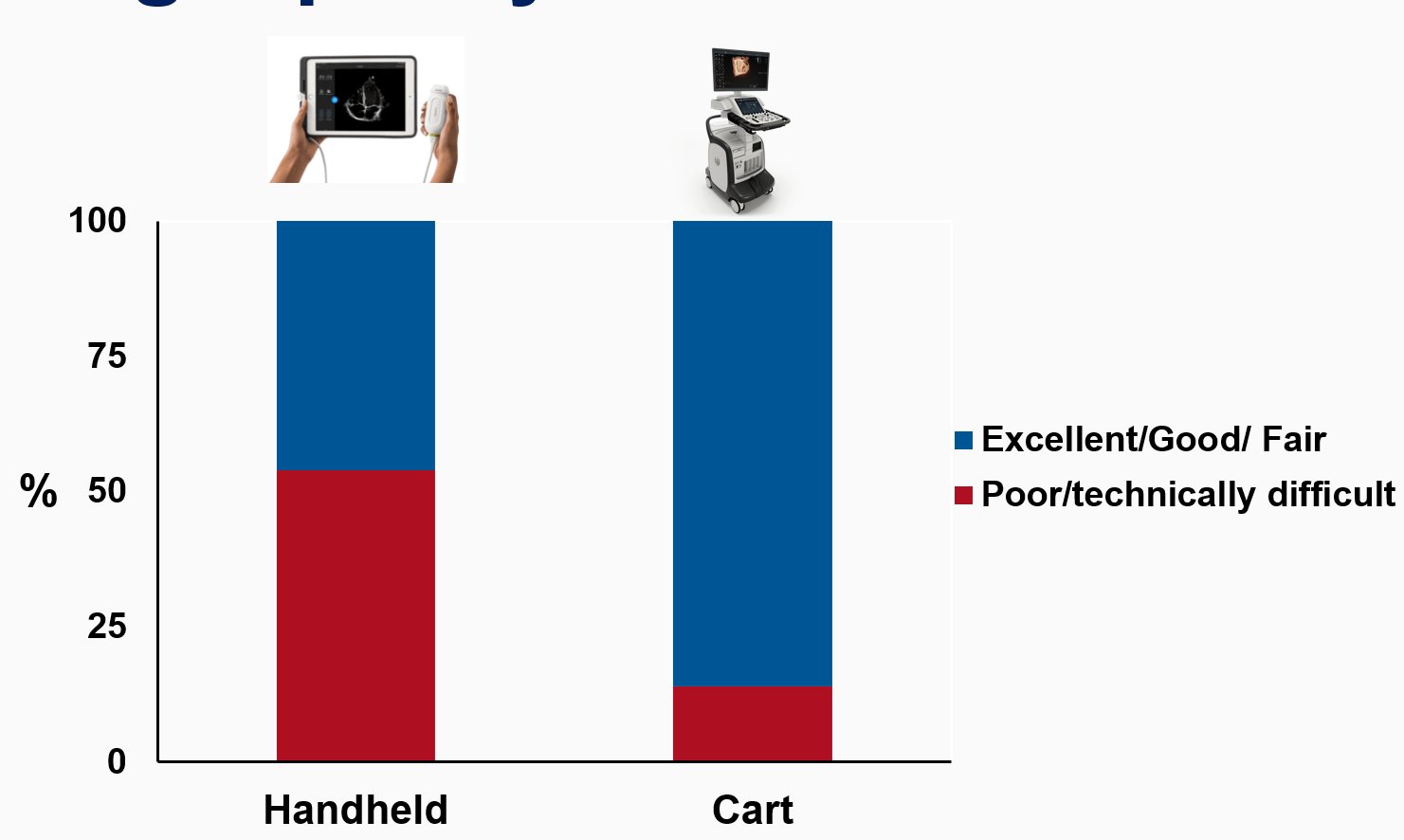


**Supplementary Figure S4**: **LVEF assessed by different modalities**


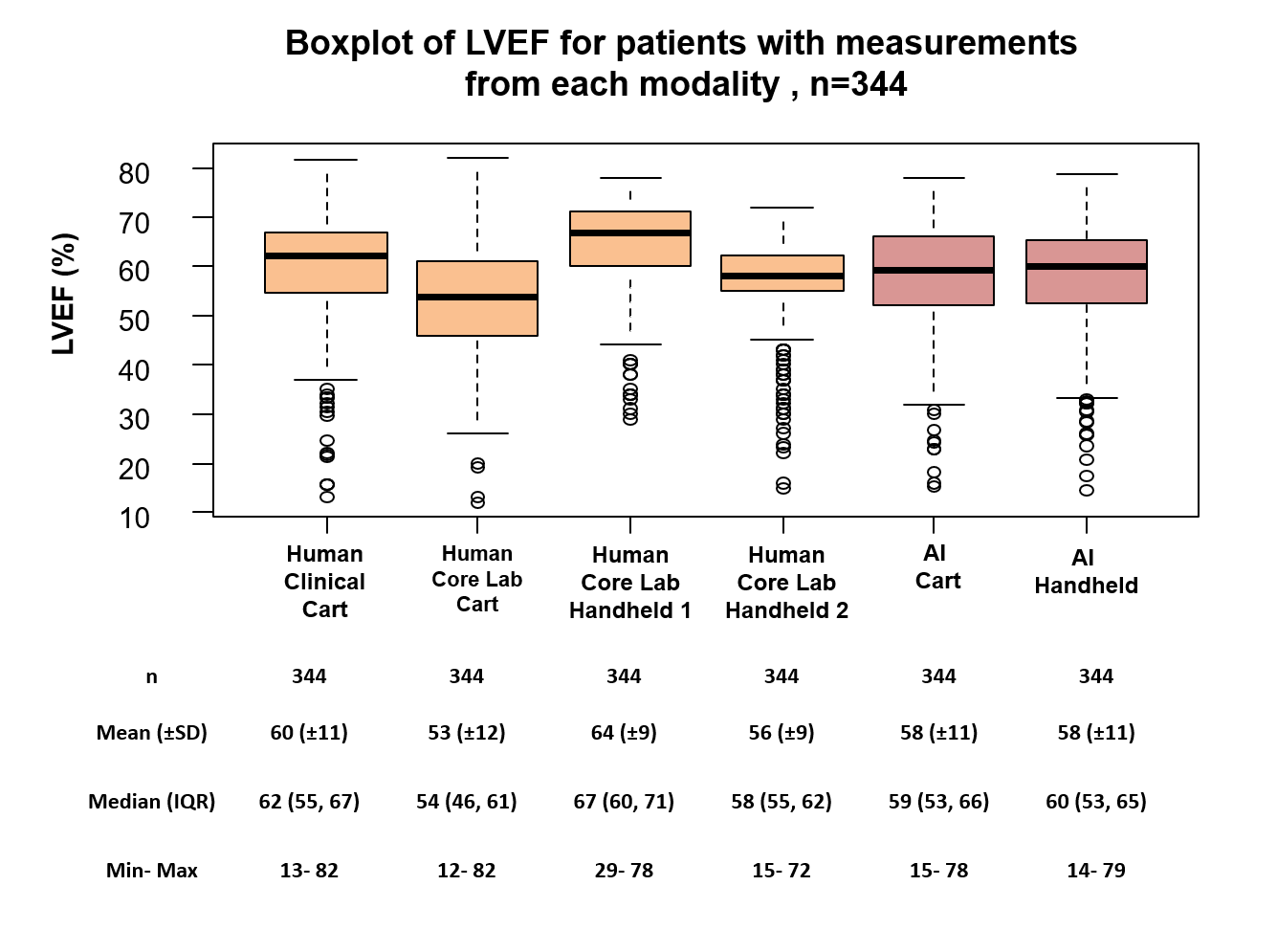


## **Supplementary figure S5-21: correlations between core laboratory and AI-automated measured LVEDV, LVIDd, LVIDs, RVIDd, TAPSE, LA volume, LV mass, MV E, MV A, MV E/A, MV dec time, E/E’, TR Vmax**

**Supplementary figure S5 Left ventricular end diastolic volume correlation and Bland-Altman plot**


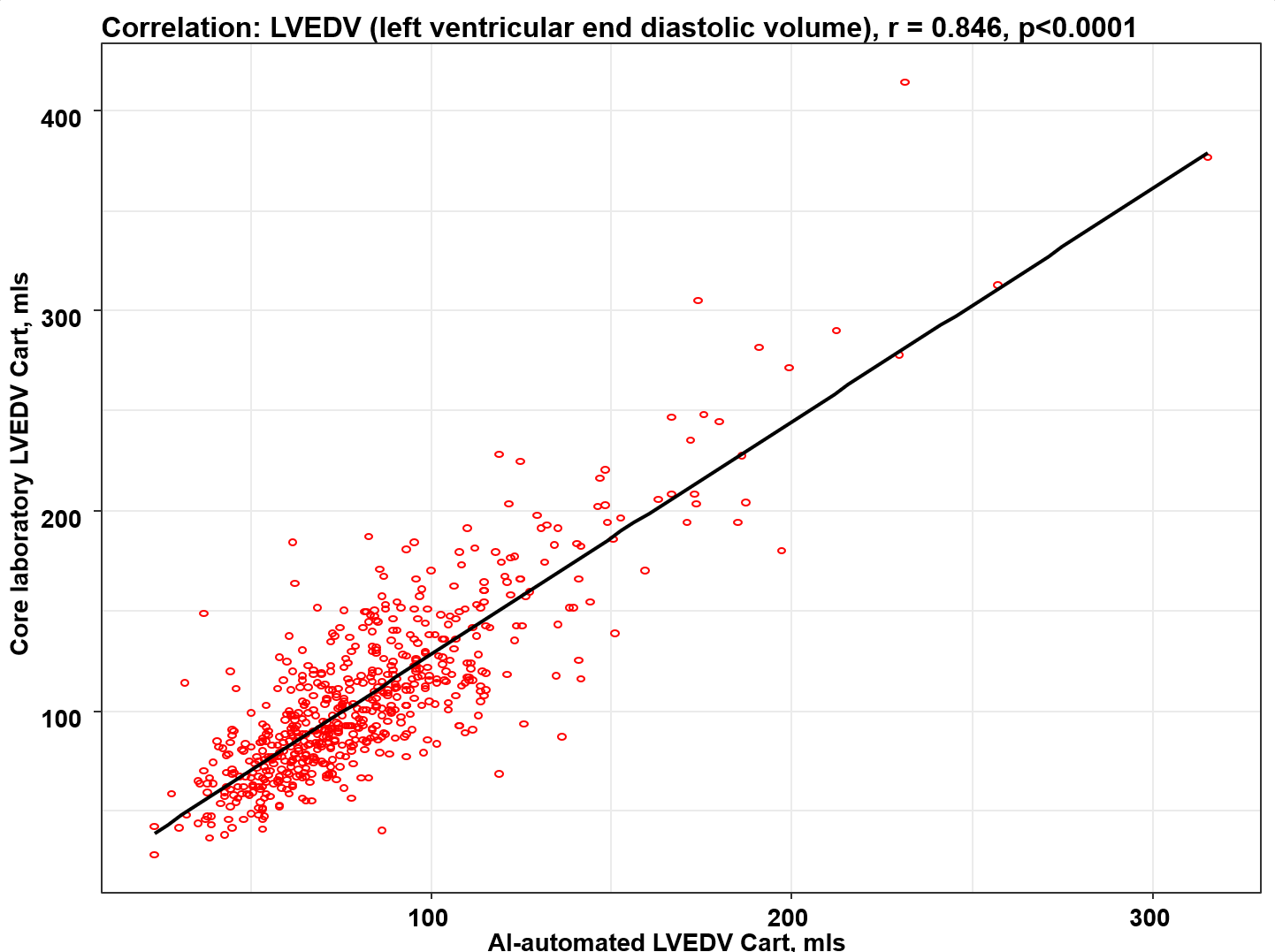


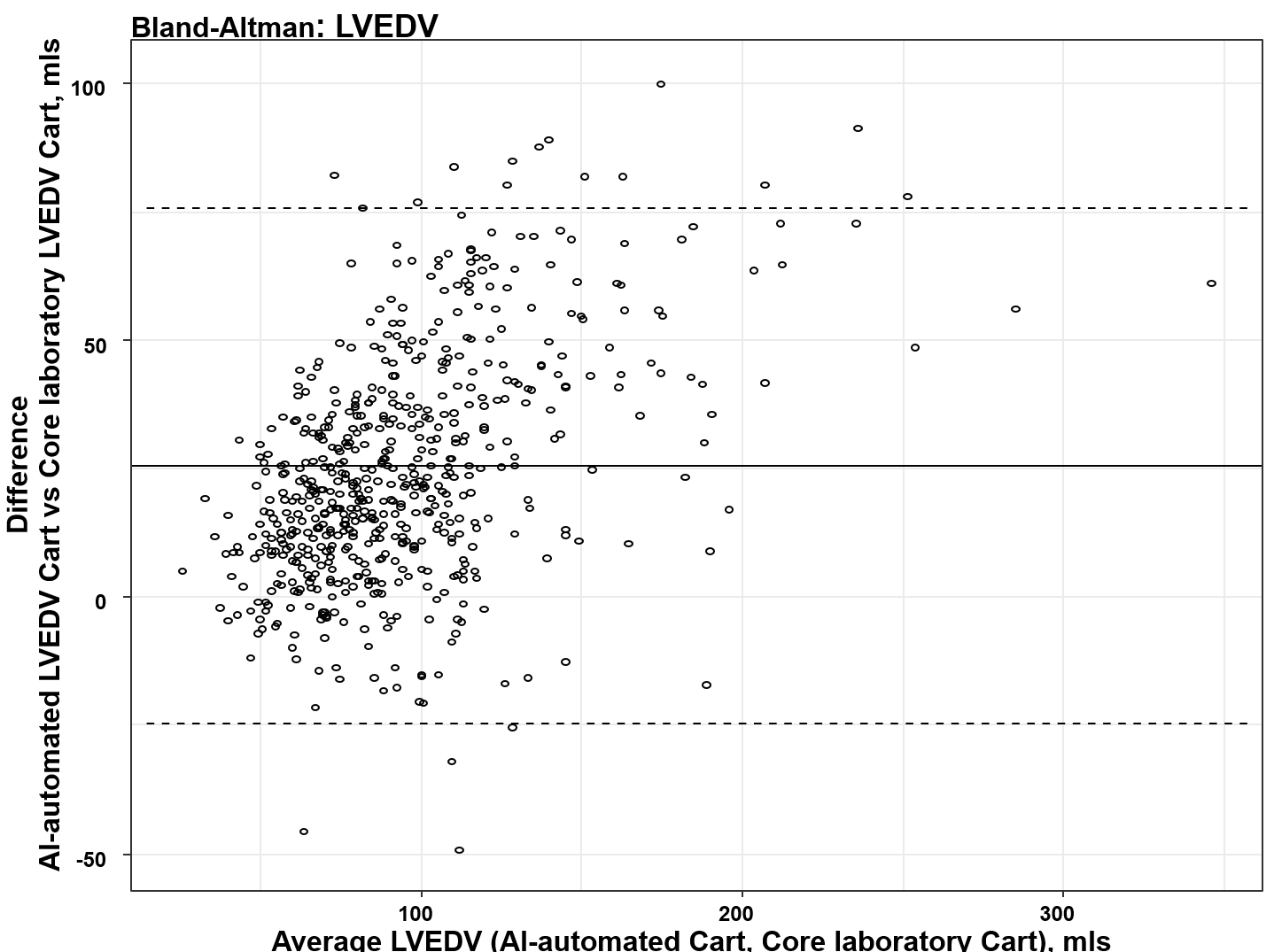


**Supplementary figure S6: Left ventricular end systolic volume correlation and Bland-Altman plot**


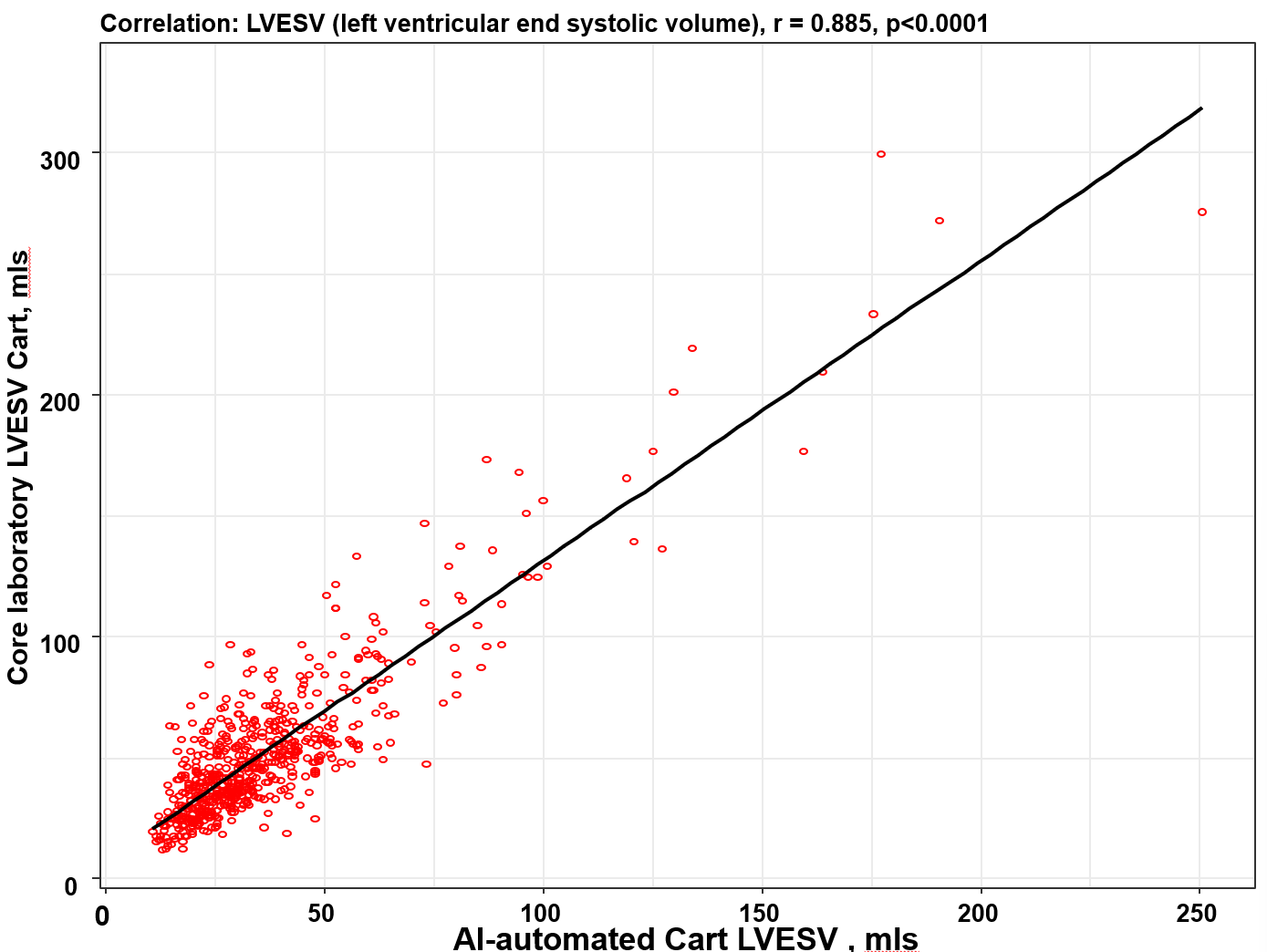


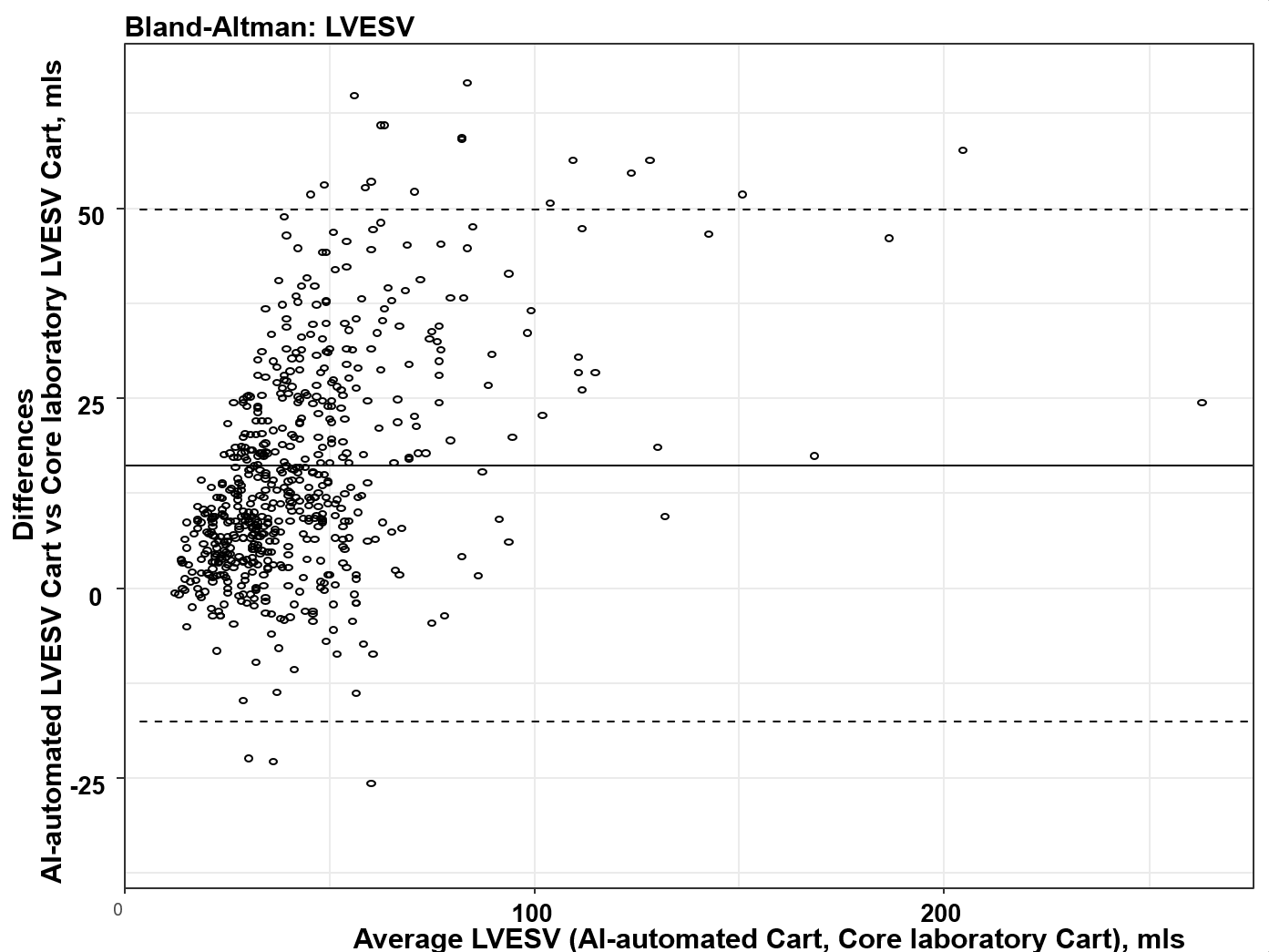


**Supplementary figure S7: Left ventricular internal diameter diastole correlation and Bland-Altman plot**


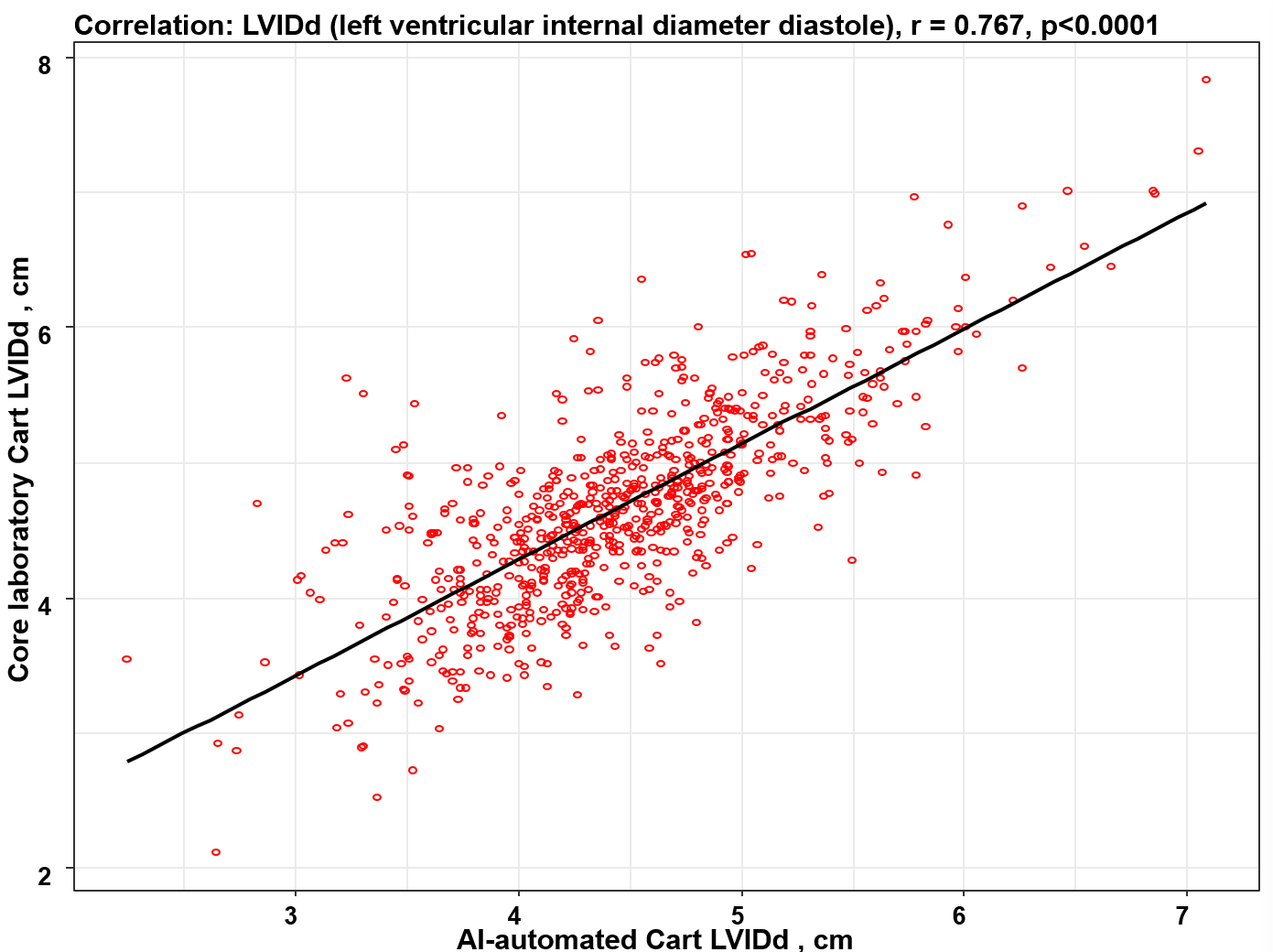


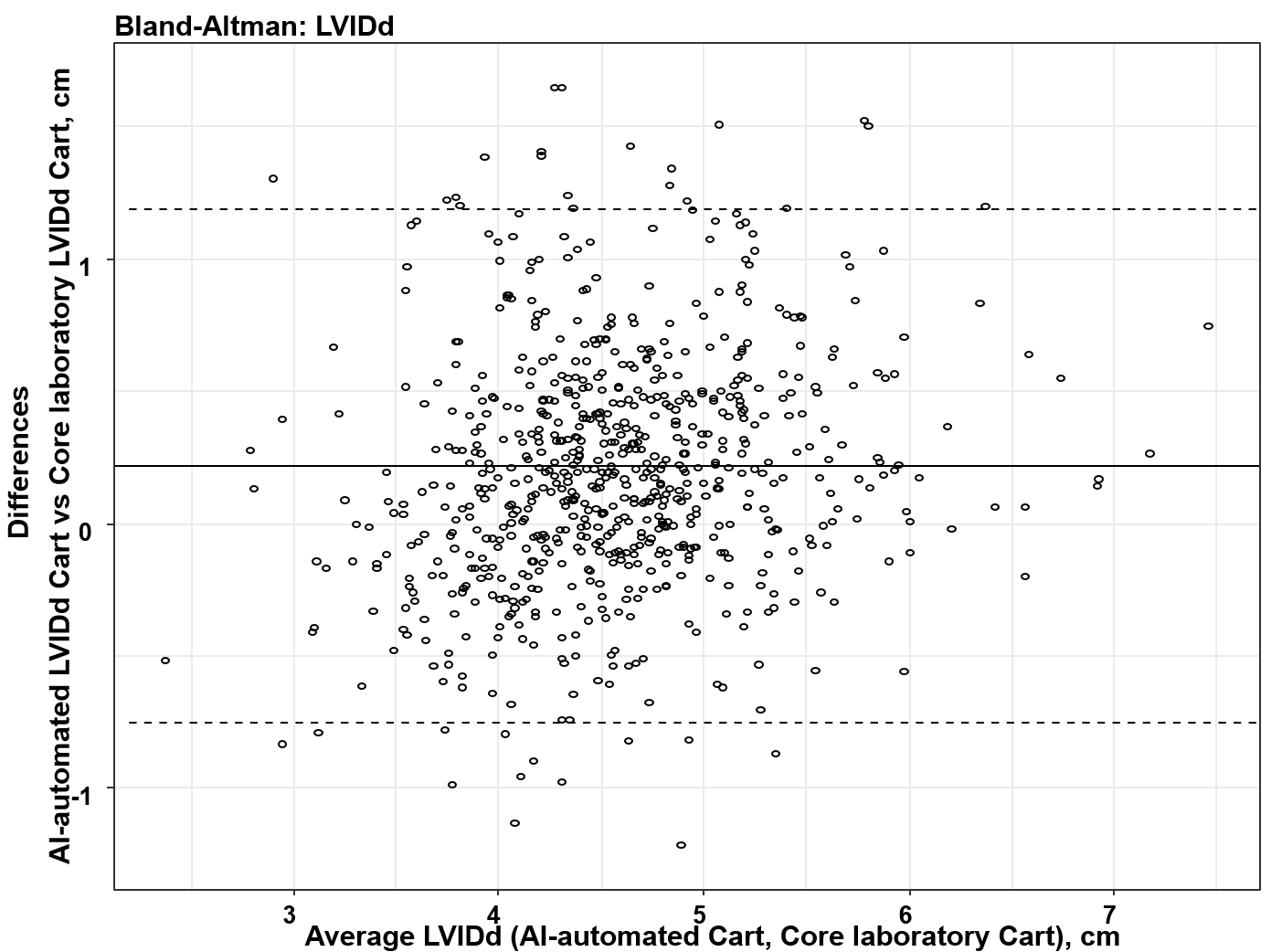


**Supplementary figure S8: Left ventricular internal diameter systole correlation and Bland-Altman plot**


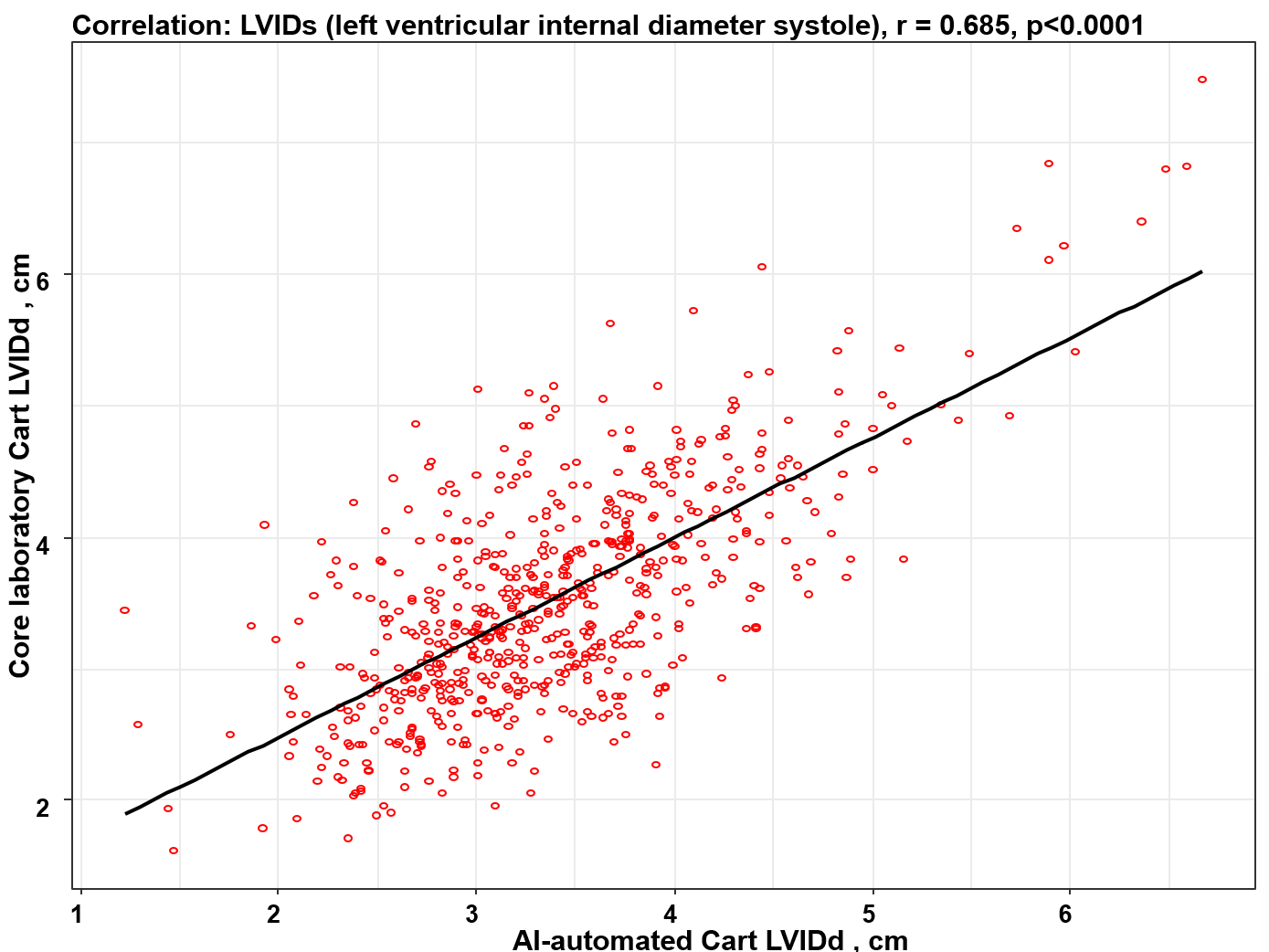


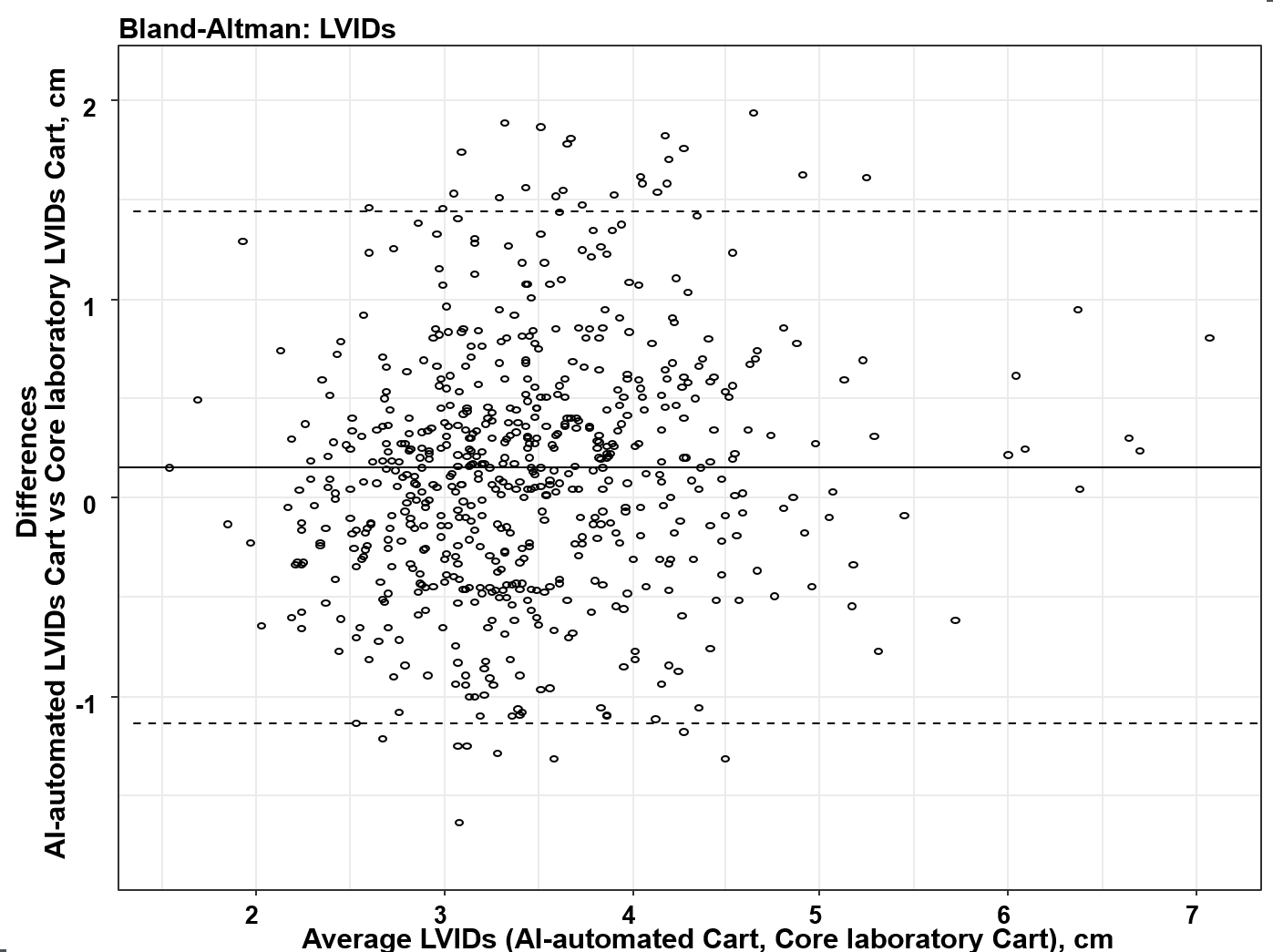


**Supplementary figure S9: Left ventricular interventricular septum diameter diastole correlation and Bland-Altman plot**


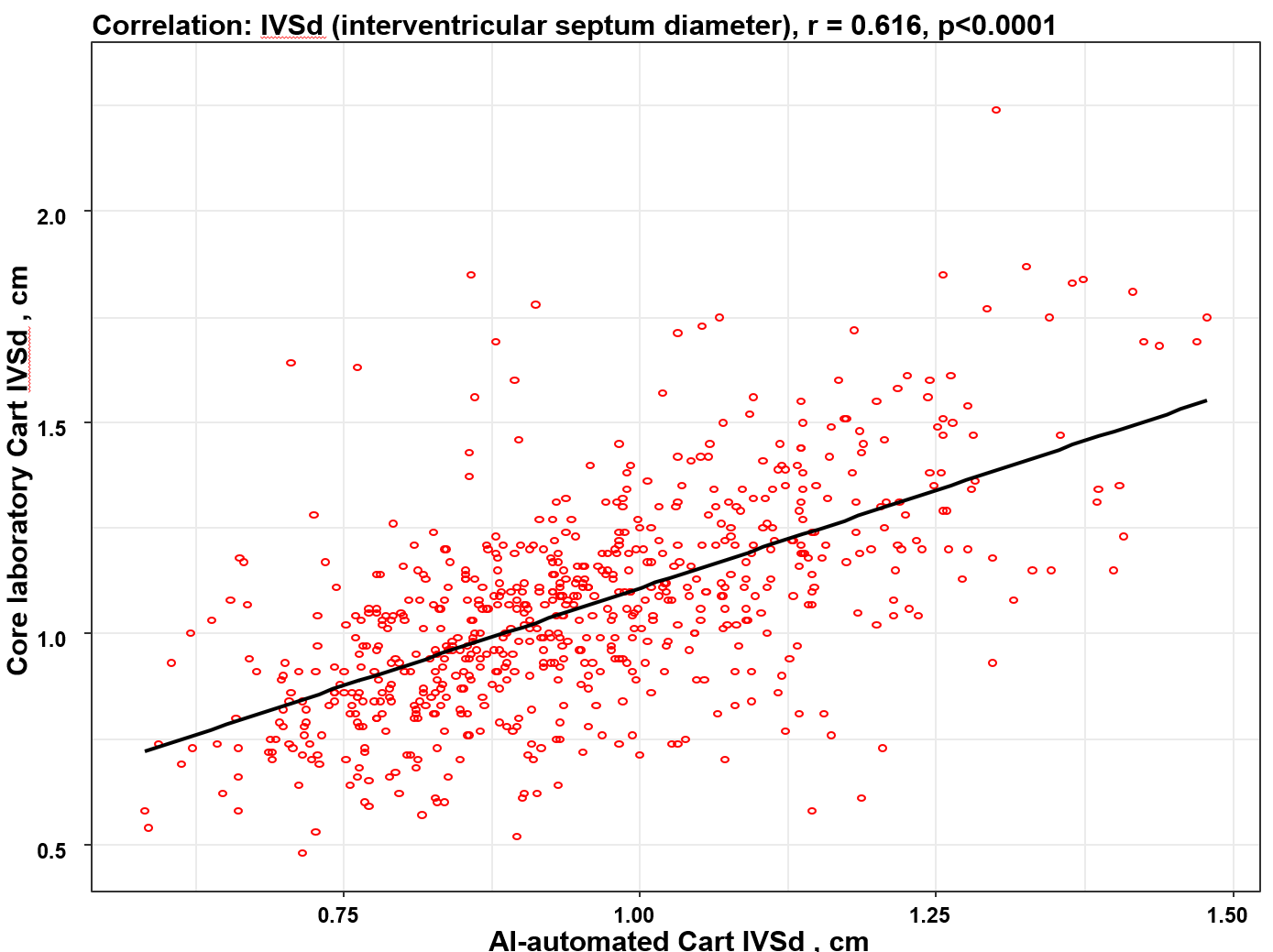


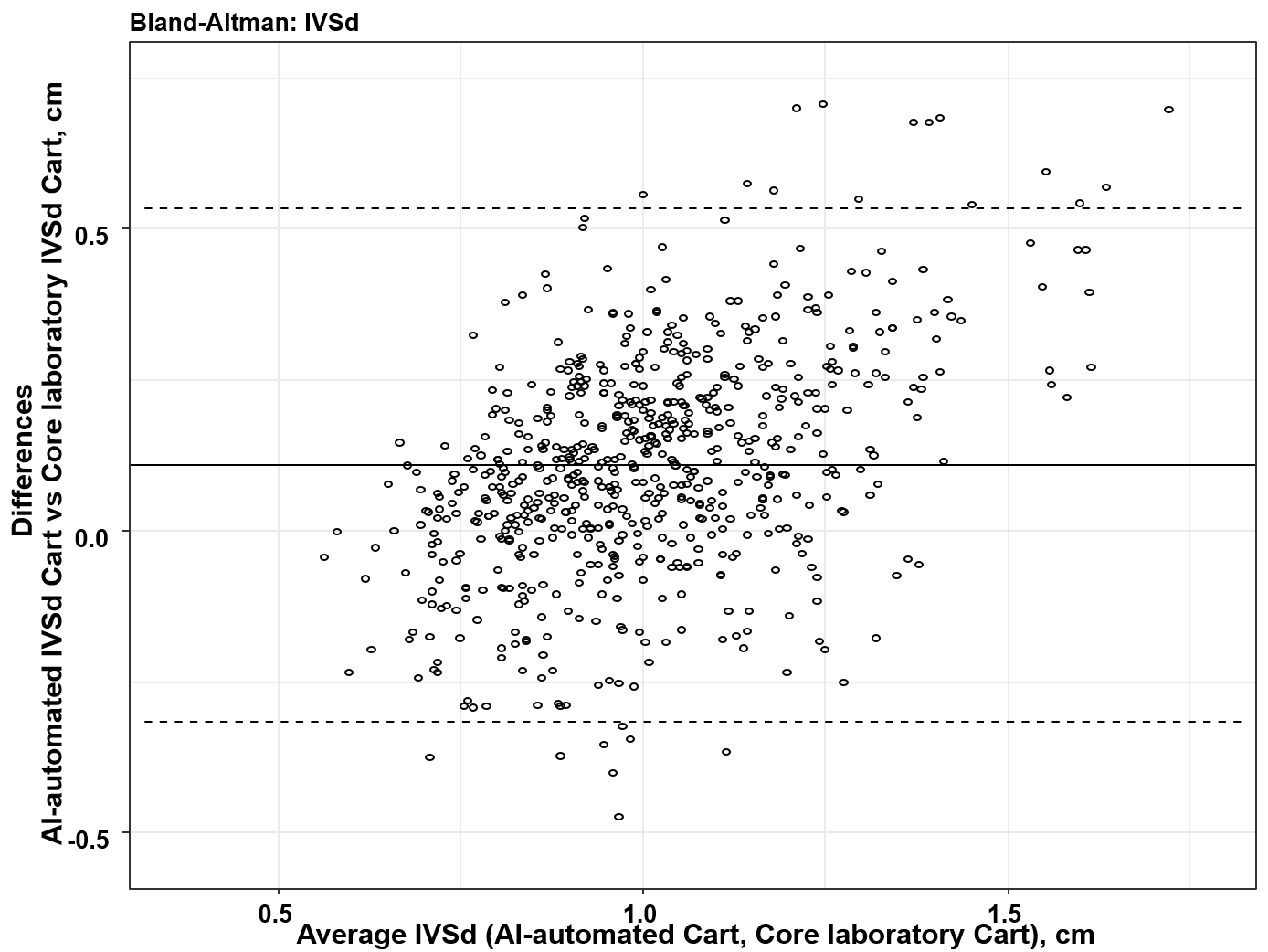


**Supplementary figure S10: Left ventricular posterior wall diameter diastole correlation and Bland-Altman plot**


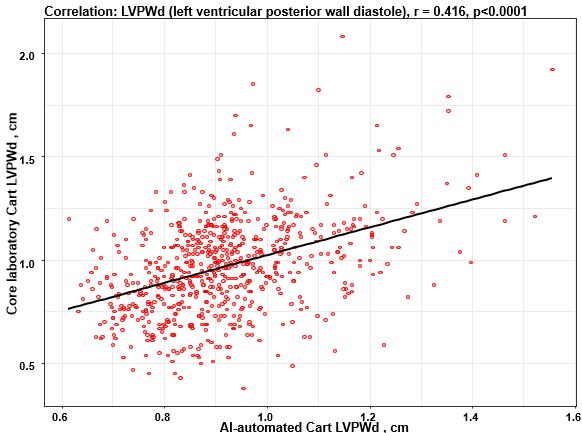


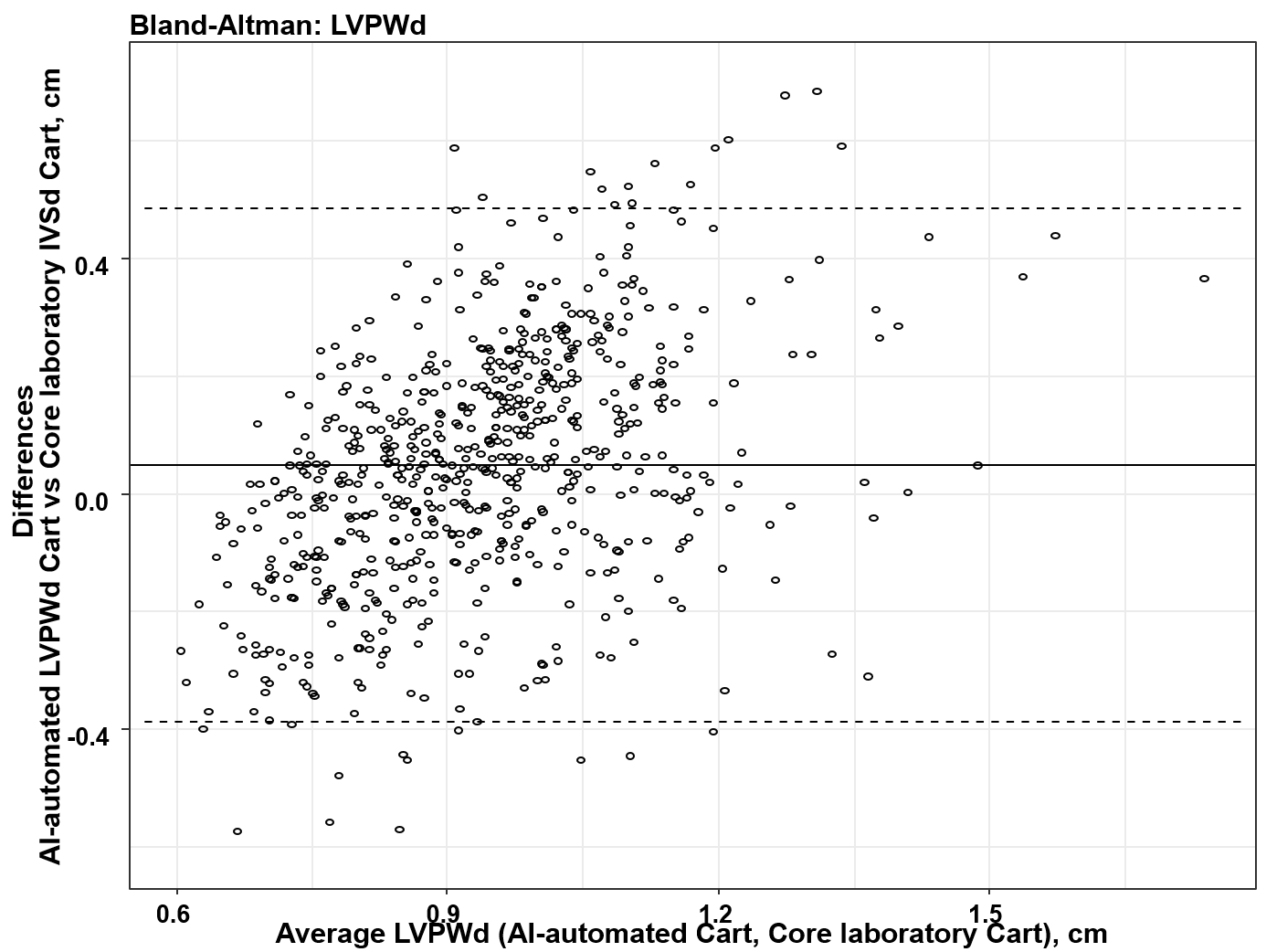


**Supplementary figure S11: Right atrial area correlation and Bland-Altman plot**


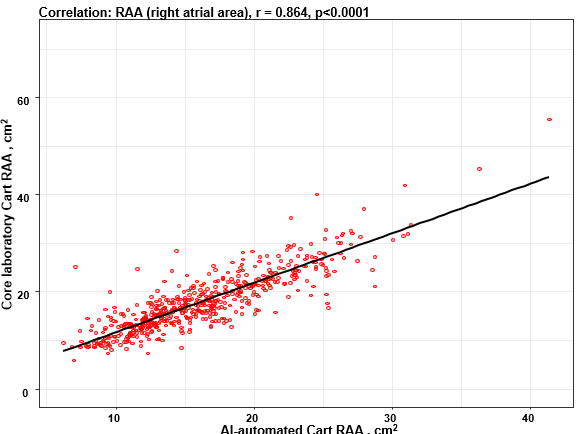


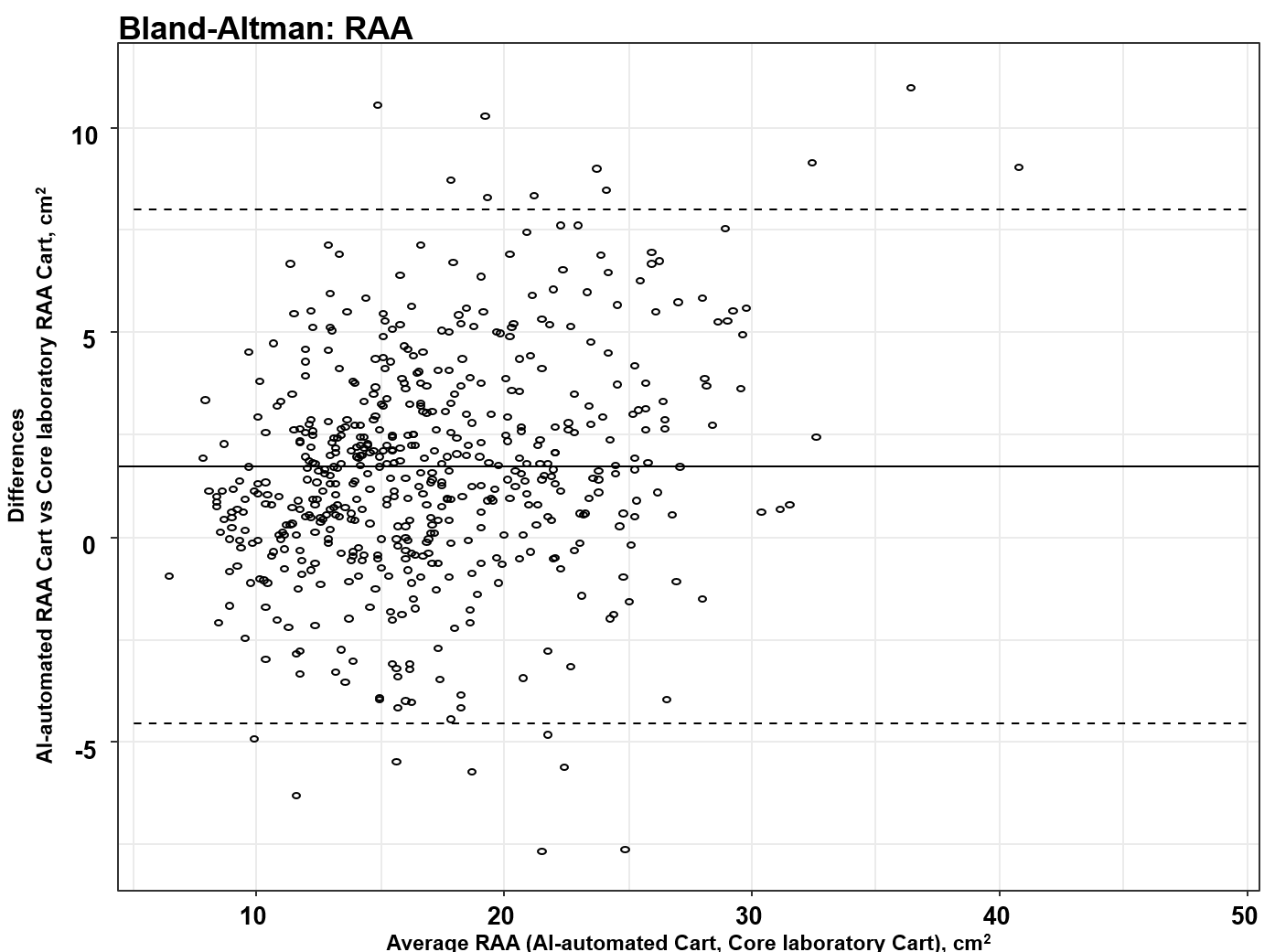


**Supplementary figure S12: Right ventricular internal diameter diastole correlation and Bland-Altman plot**


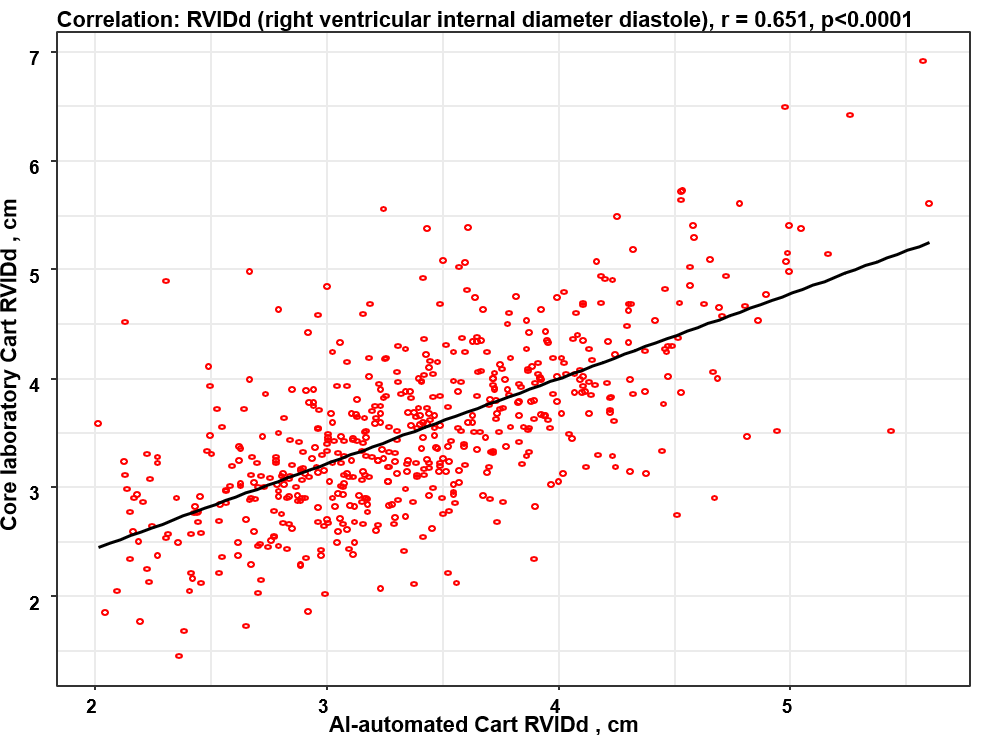


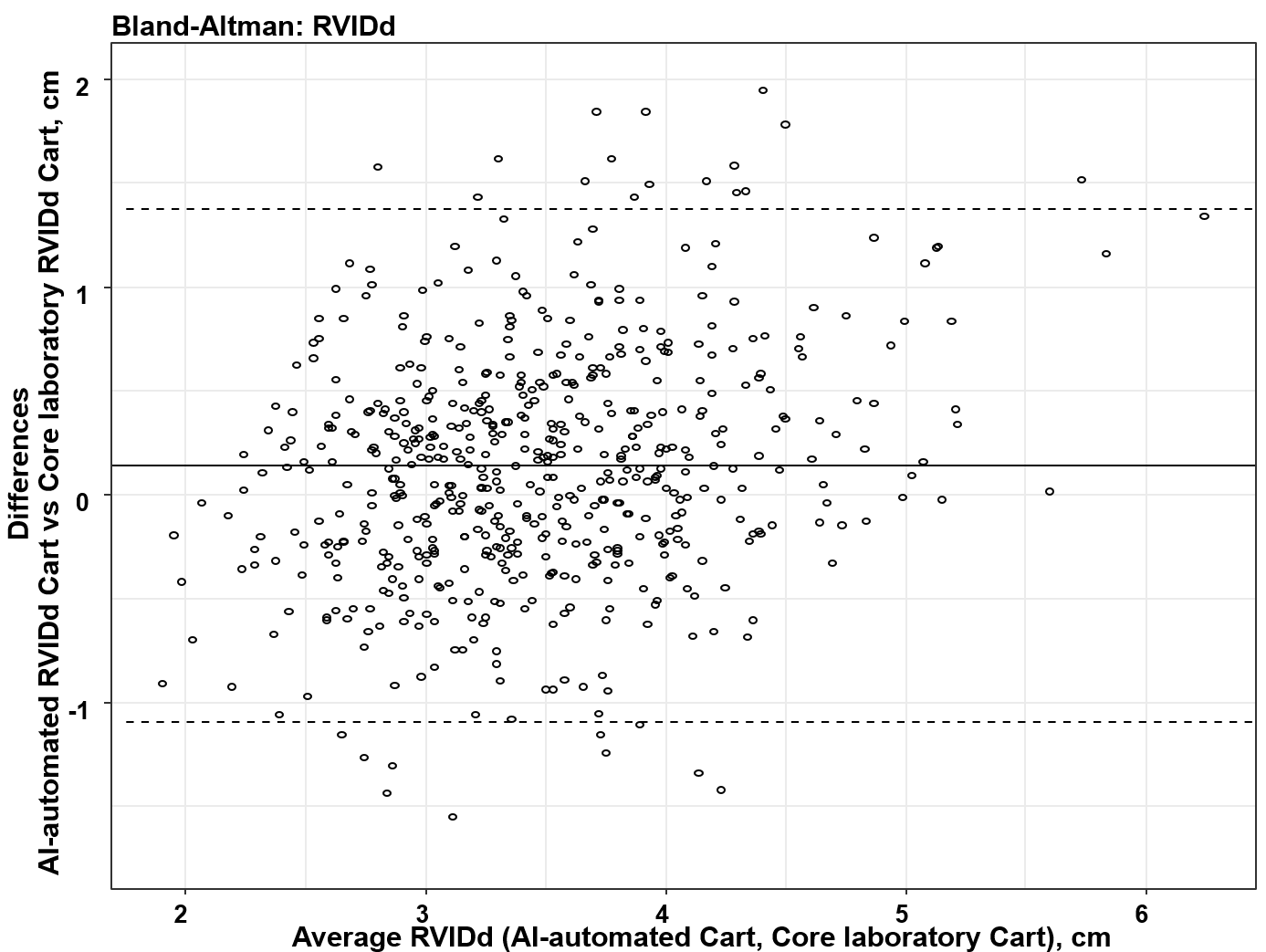


**Supplementary figure S13: Tricuspid annular plane systolic excursion correlation and Bland-Altman plot**


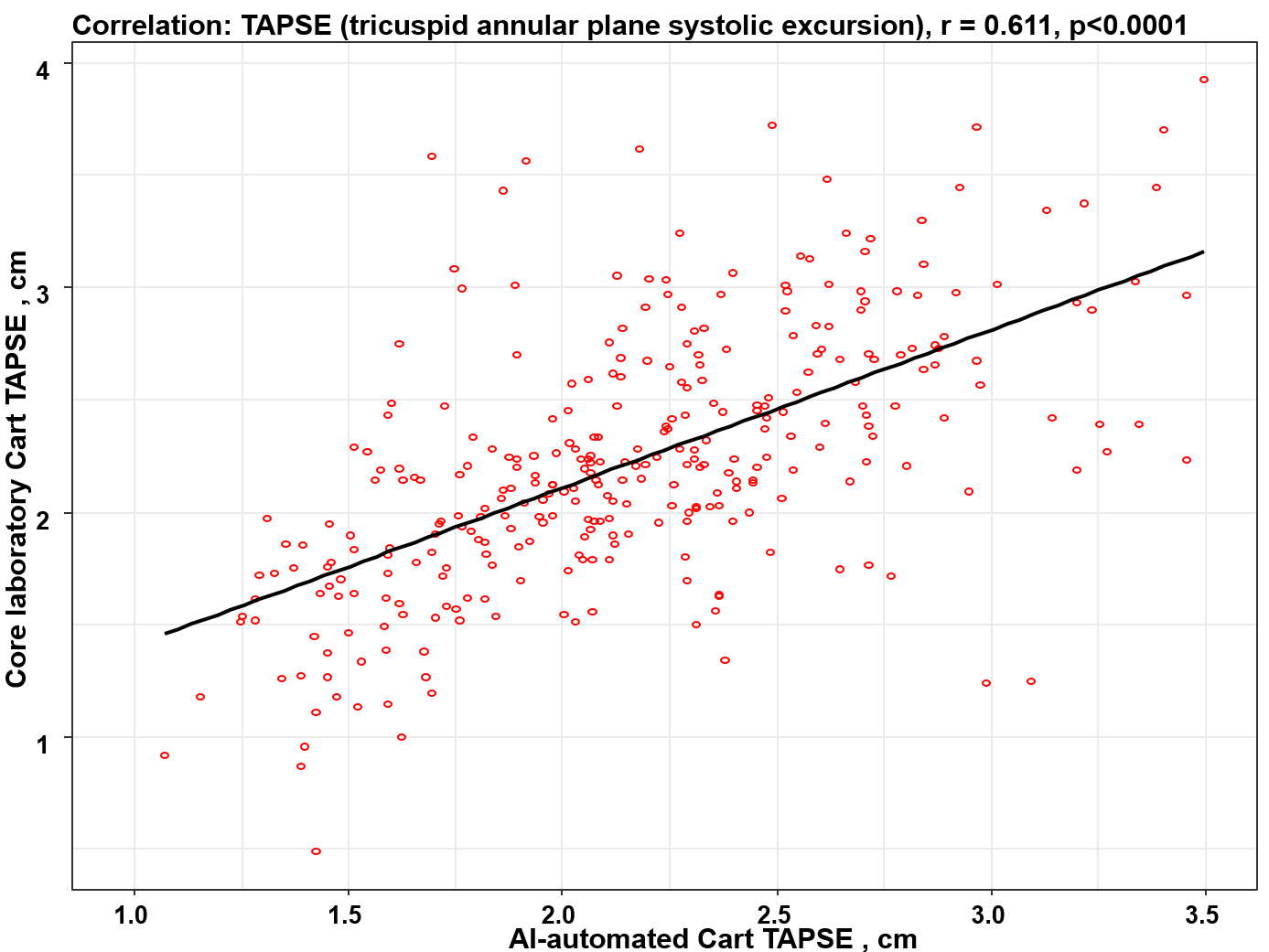


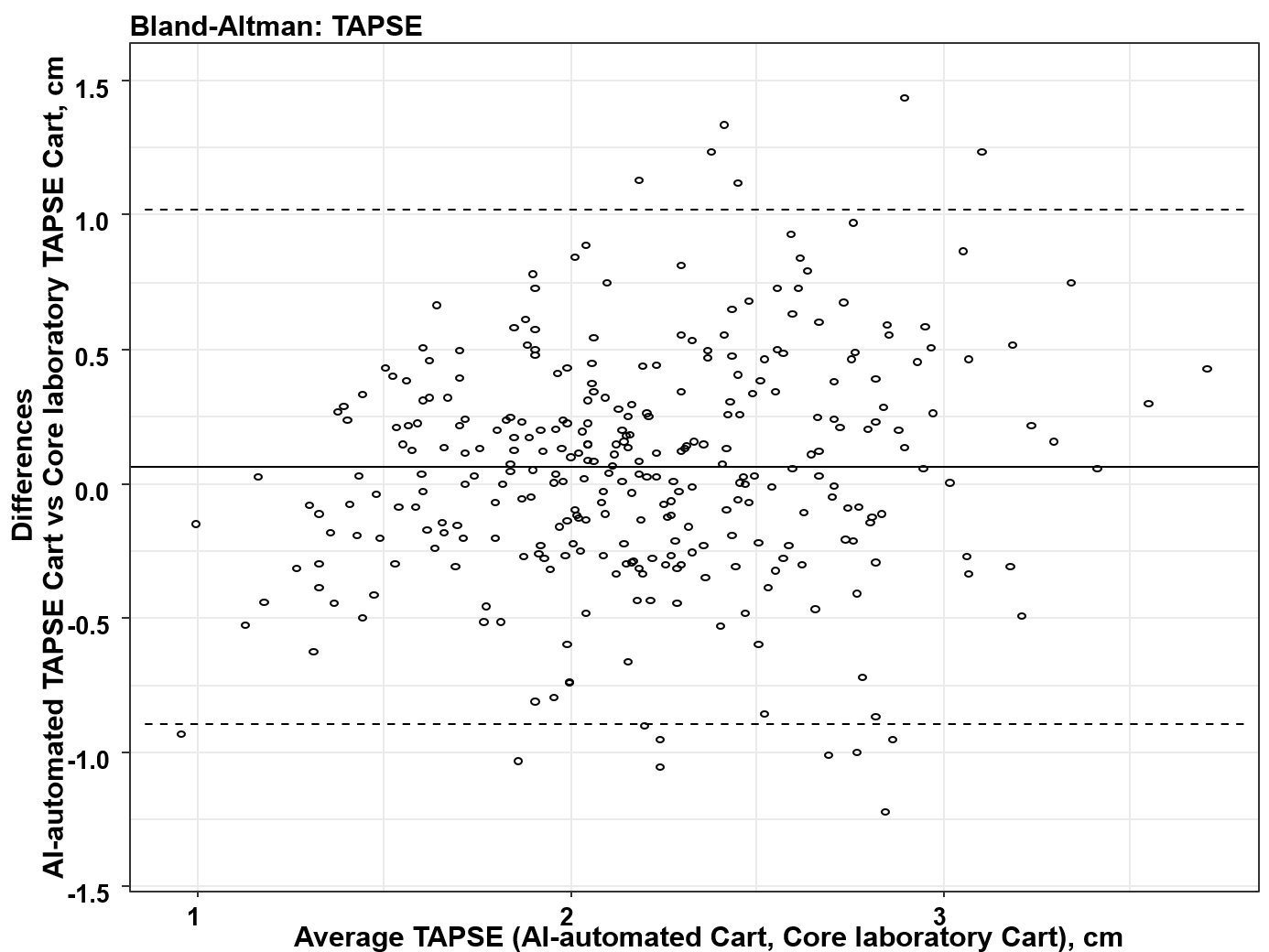


**Supplementary figure S14: Left atrial volume correlation and Bland-Altman plot**


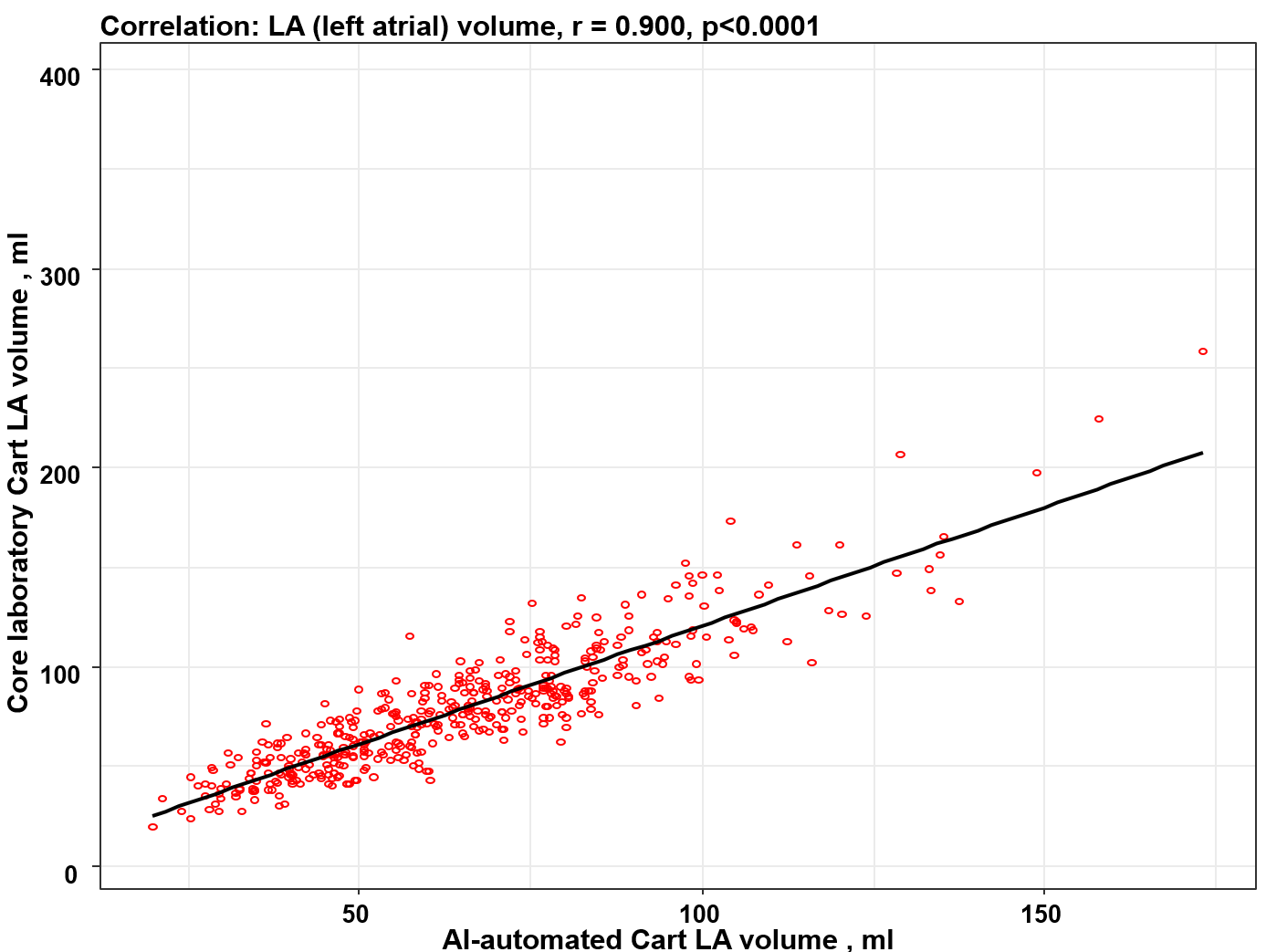


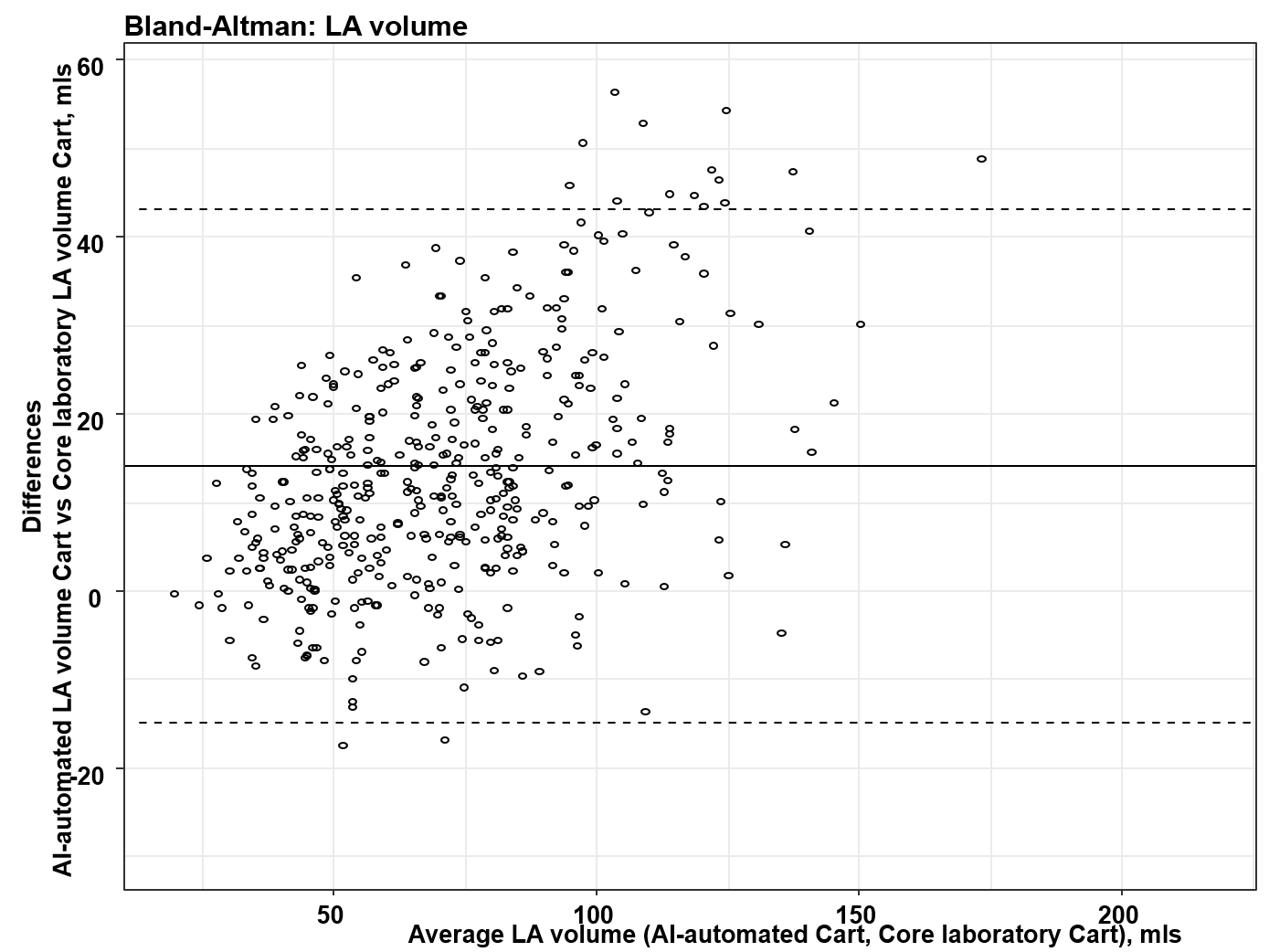


**Supplementary figure S15: Left ventricular mass correlation and Bland-Altman plot**


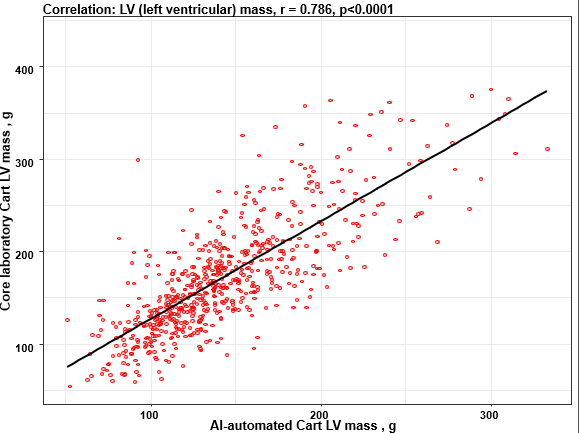


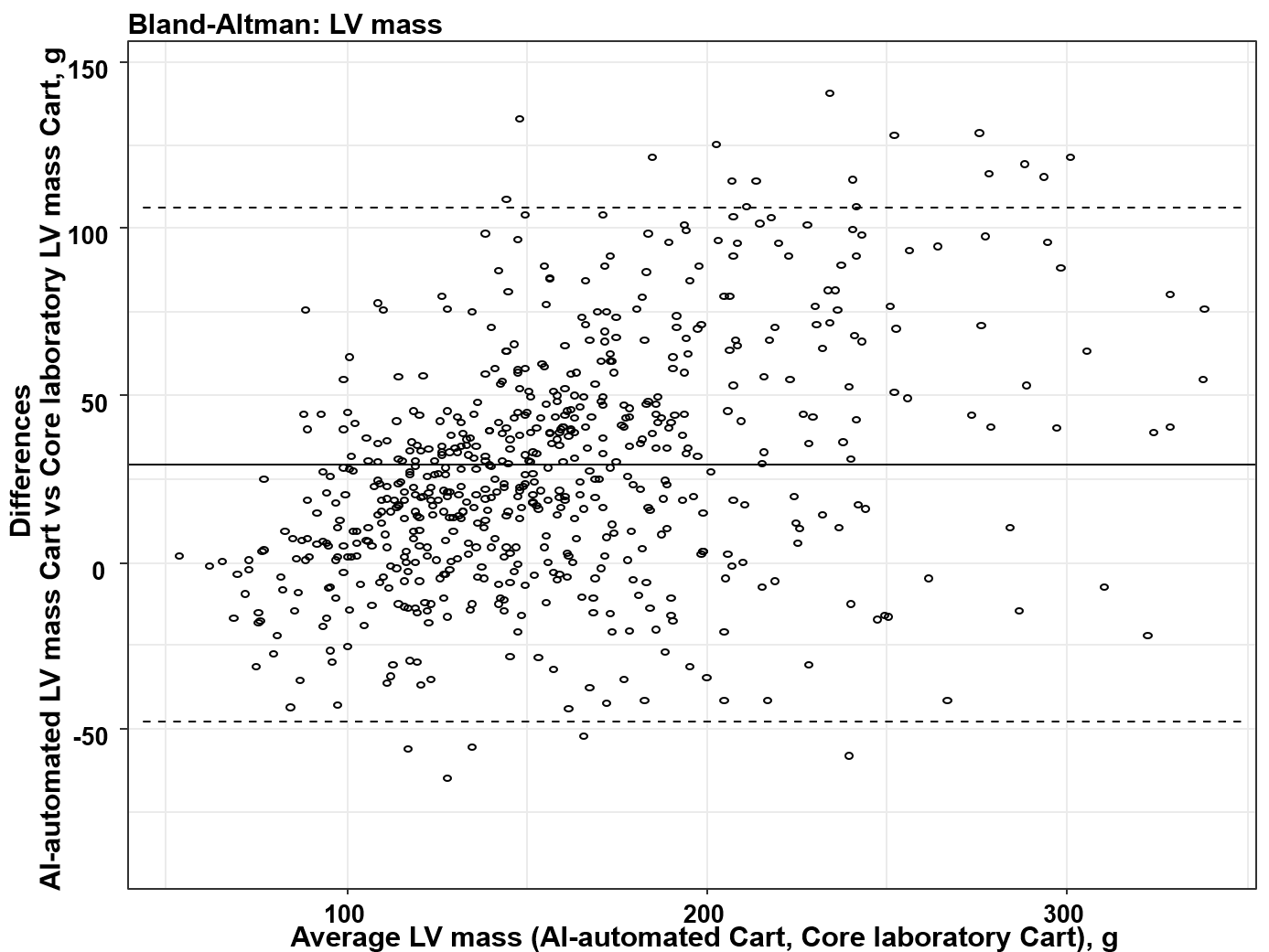


**Supplementary figure S16: mitral valve E velocity correlation and Bland-Altman plot**


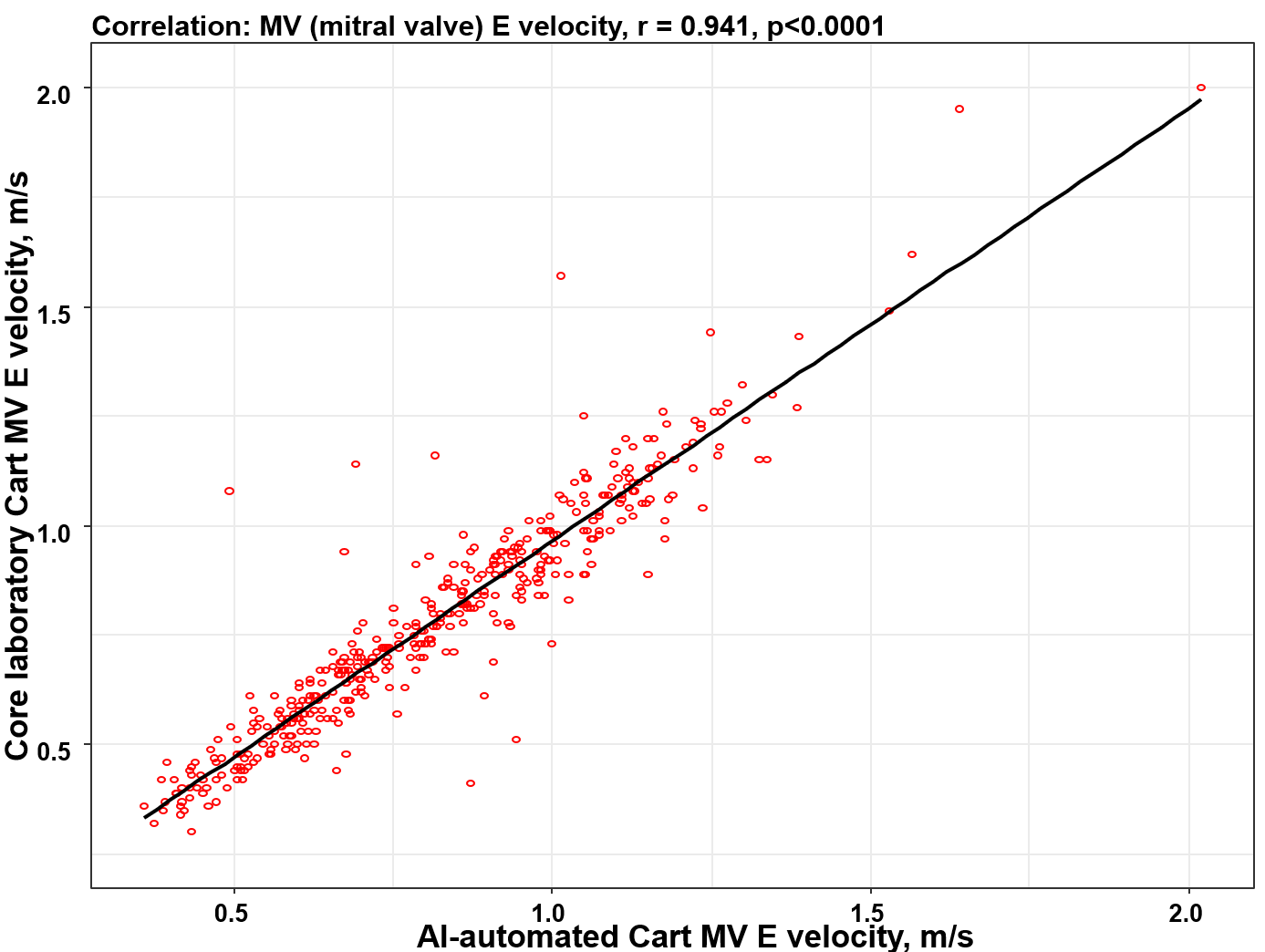


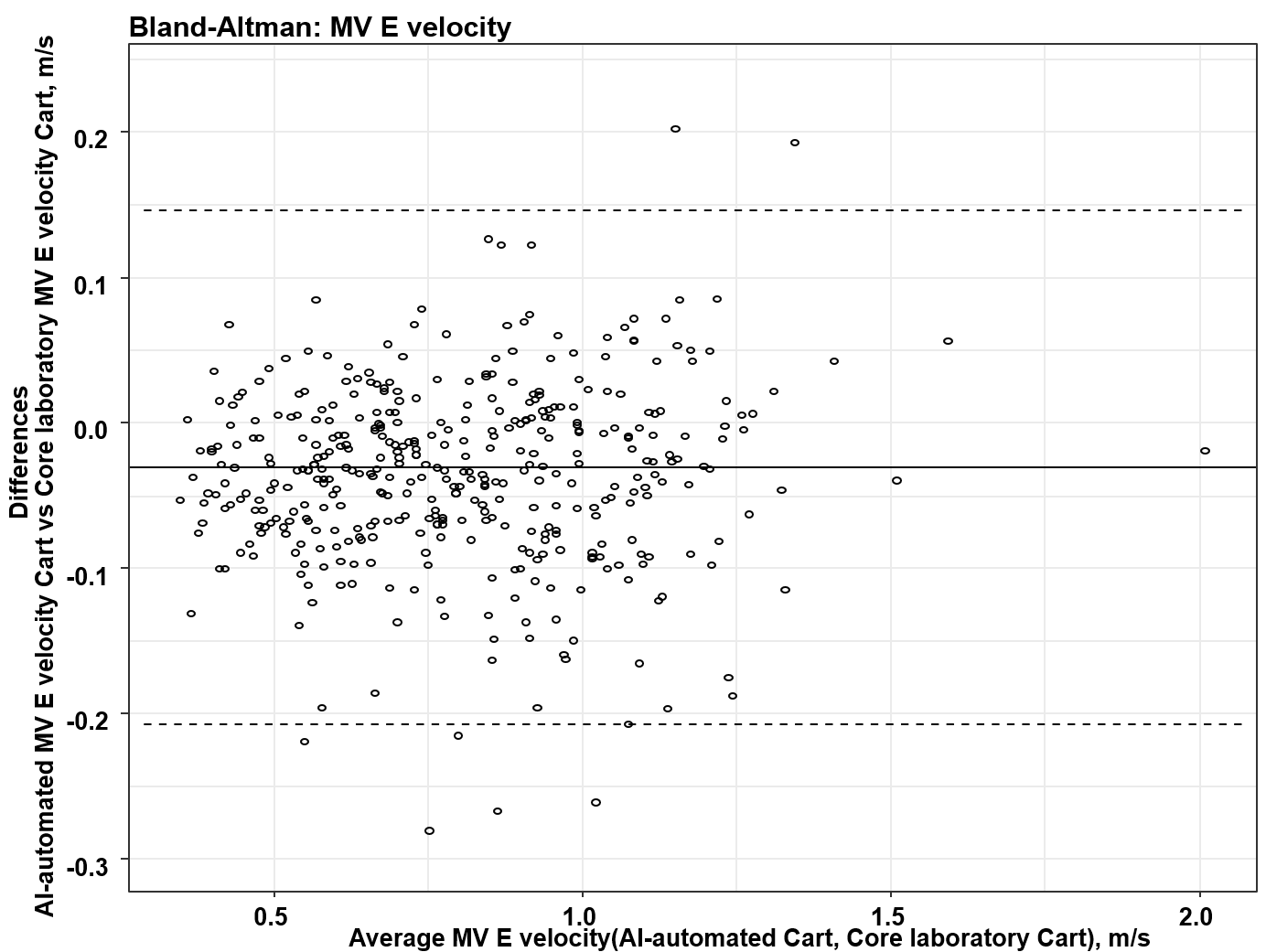


**Supplementary figure S17: mitral valve A velocity correlation and Bland-Altman plot**


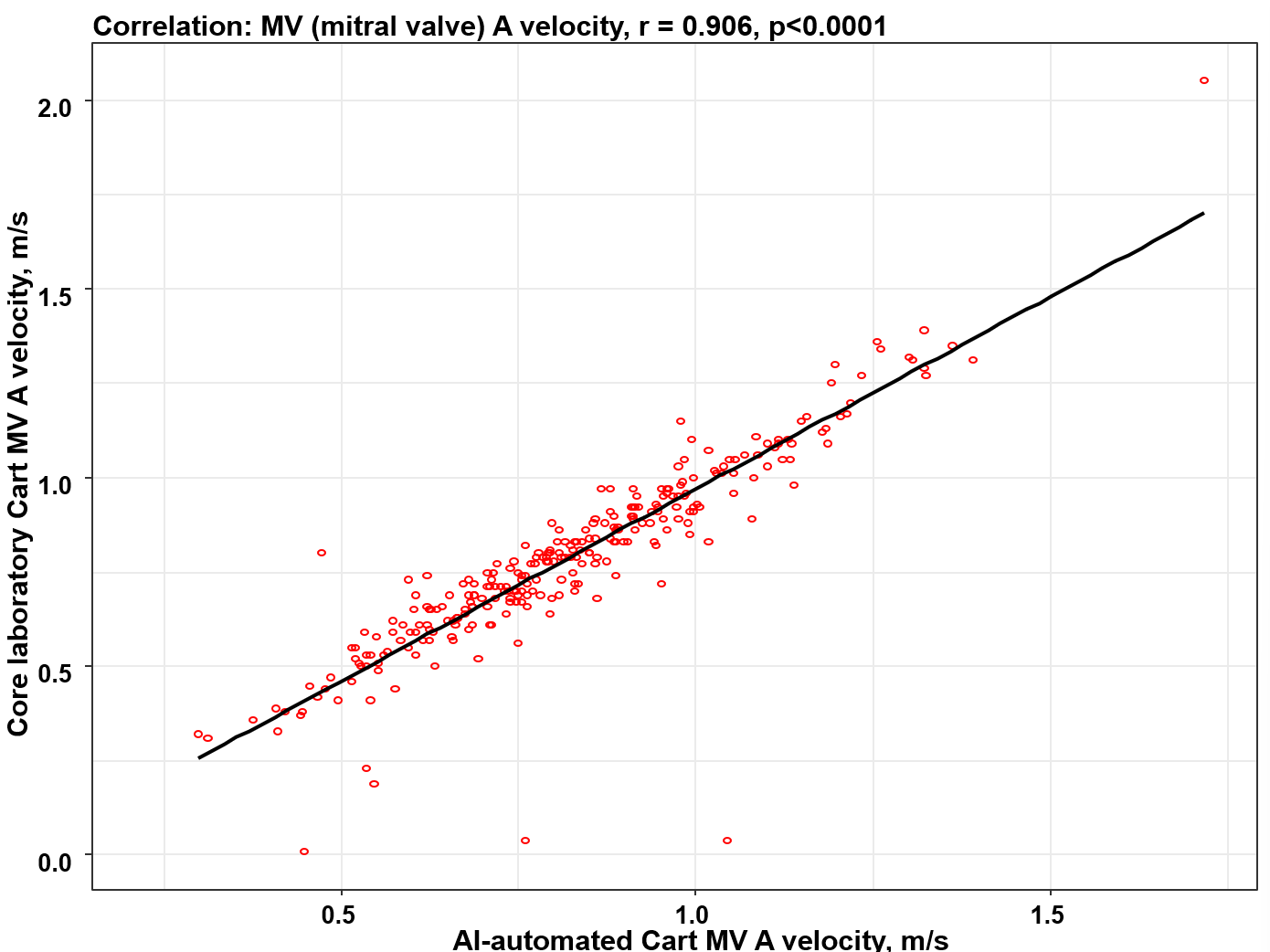


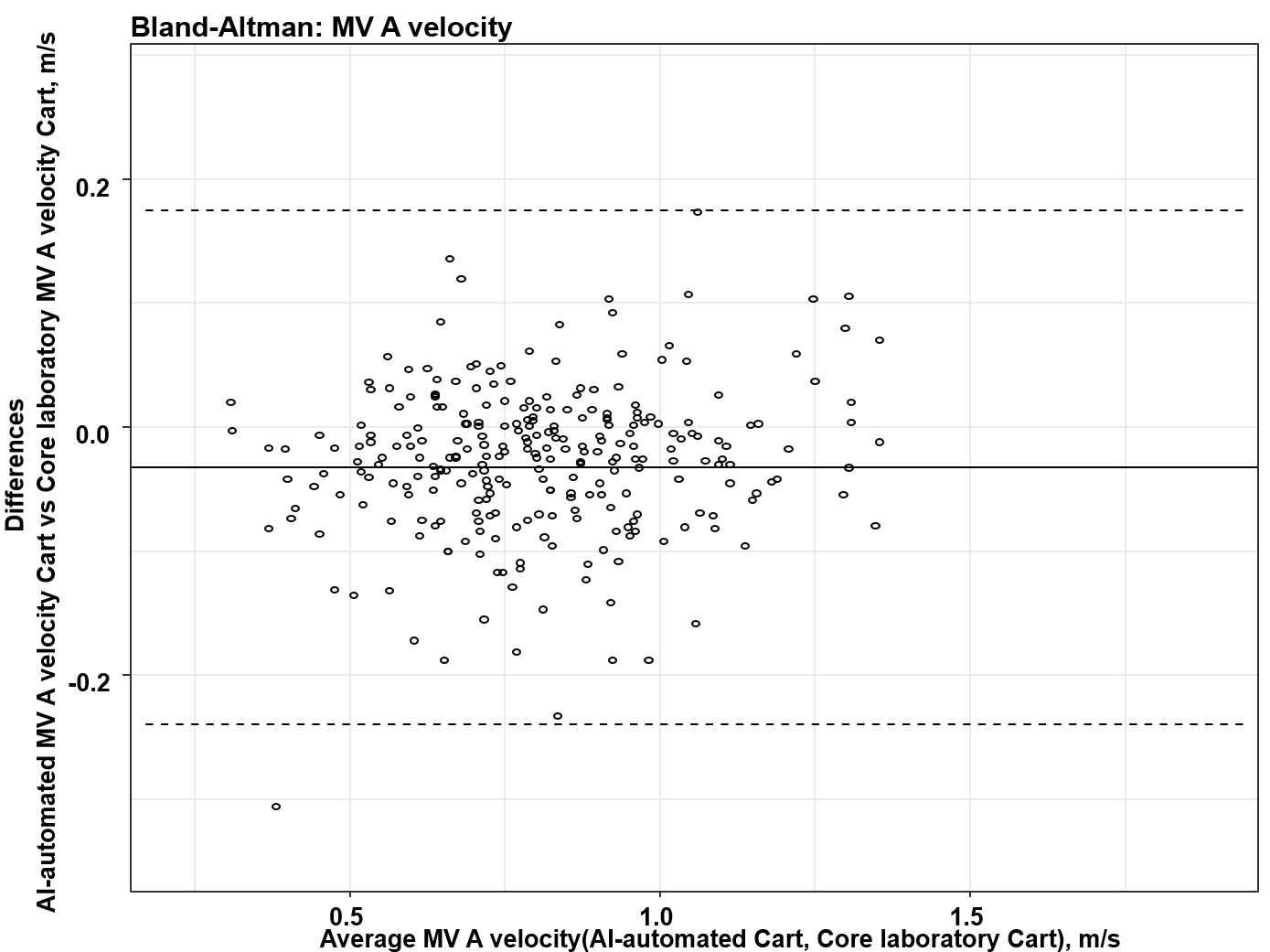


**Supplementary figure S18: mitral valve E/A ratio correlation and Bland-Altman plot**


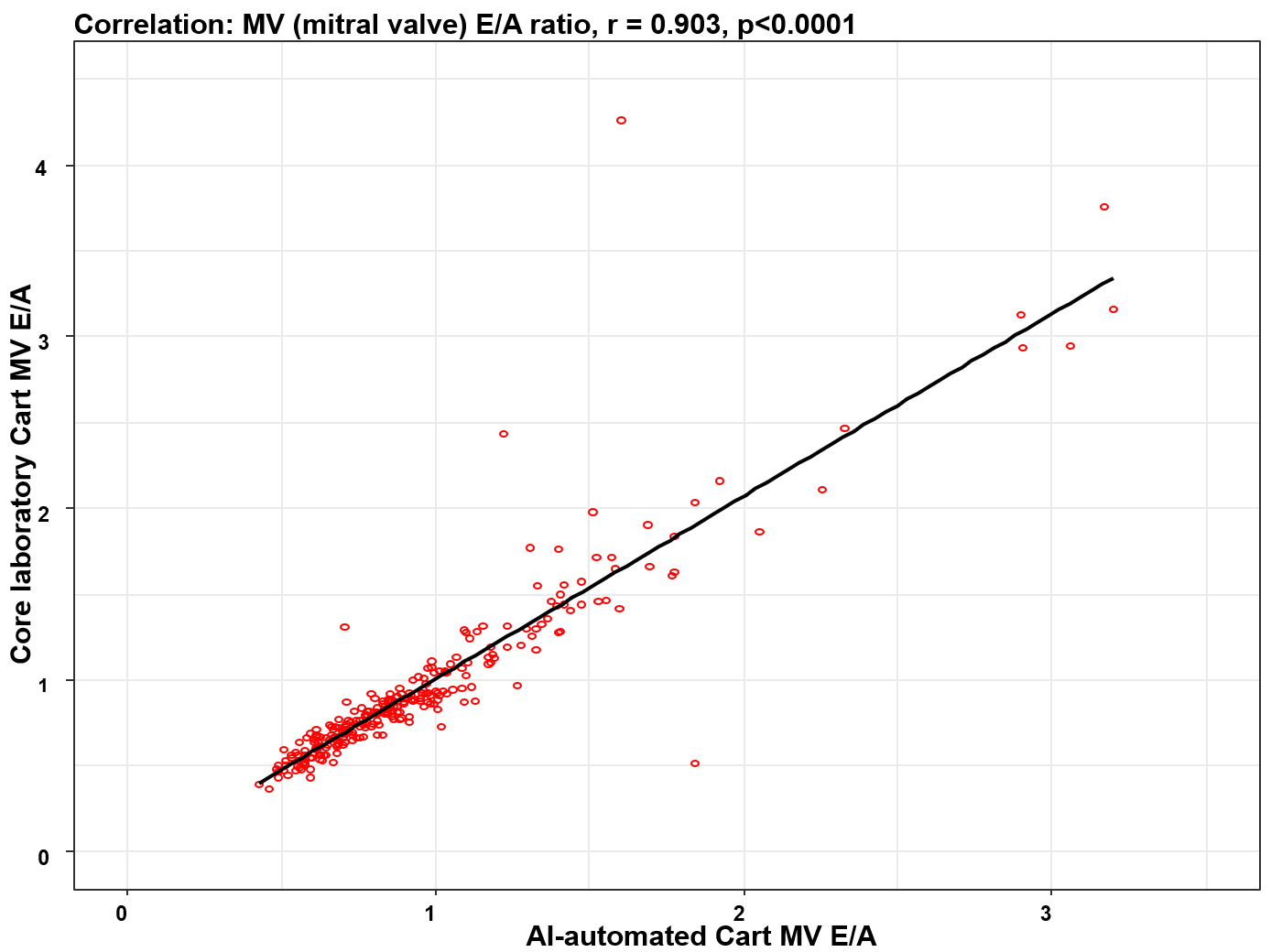


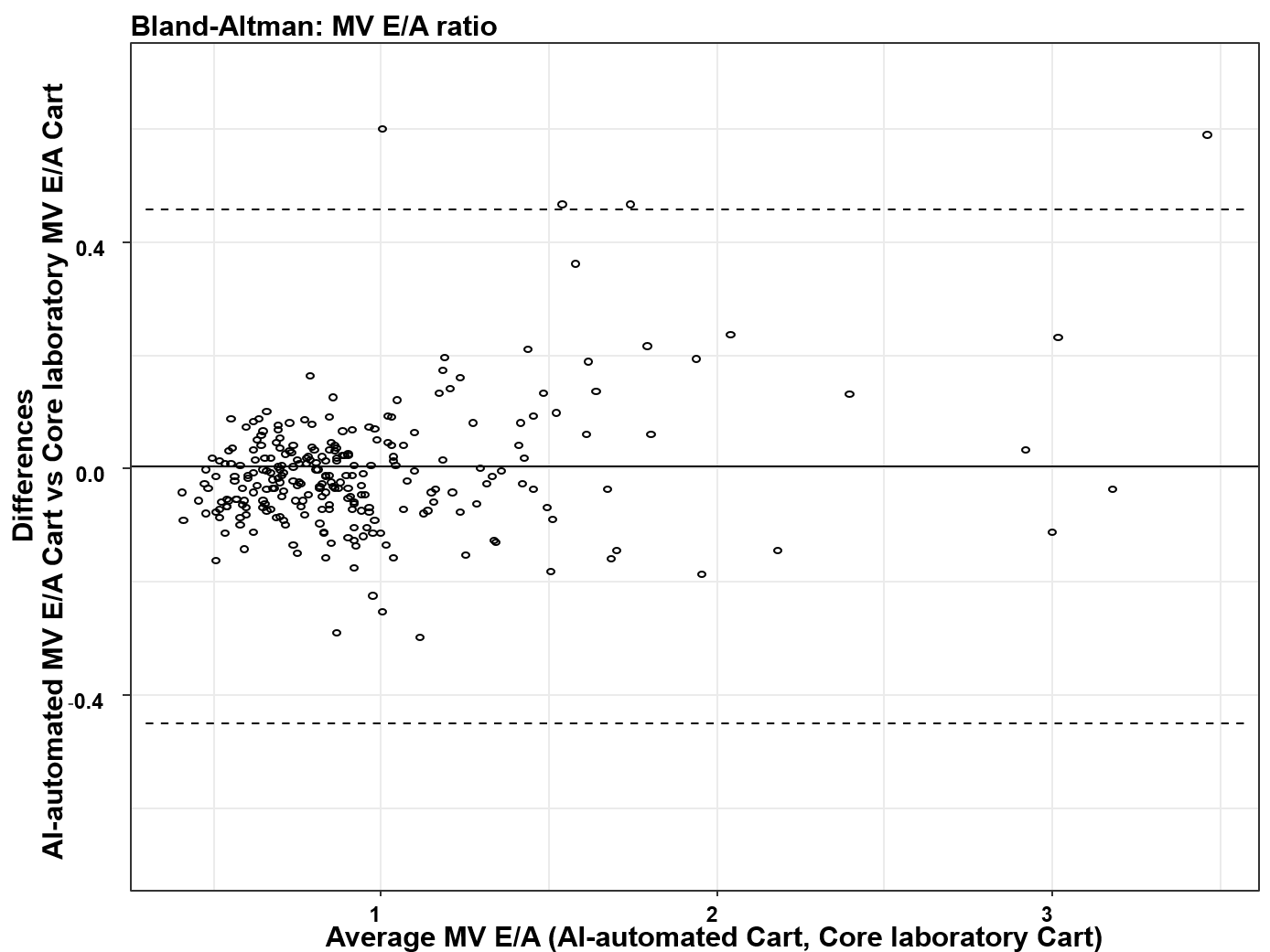


**Supplementary figure S19: mitral valve deceleration time correlation and Bland-Altman plot**


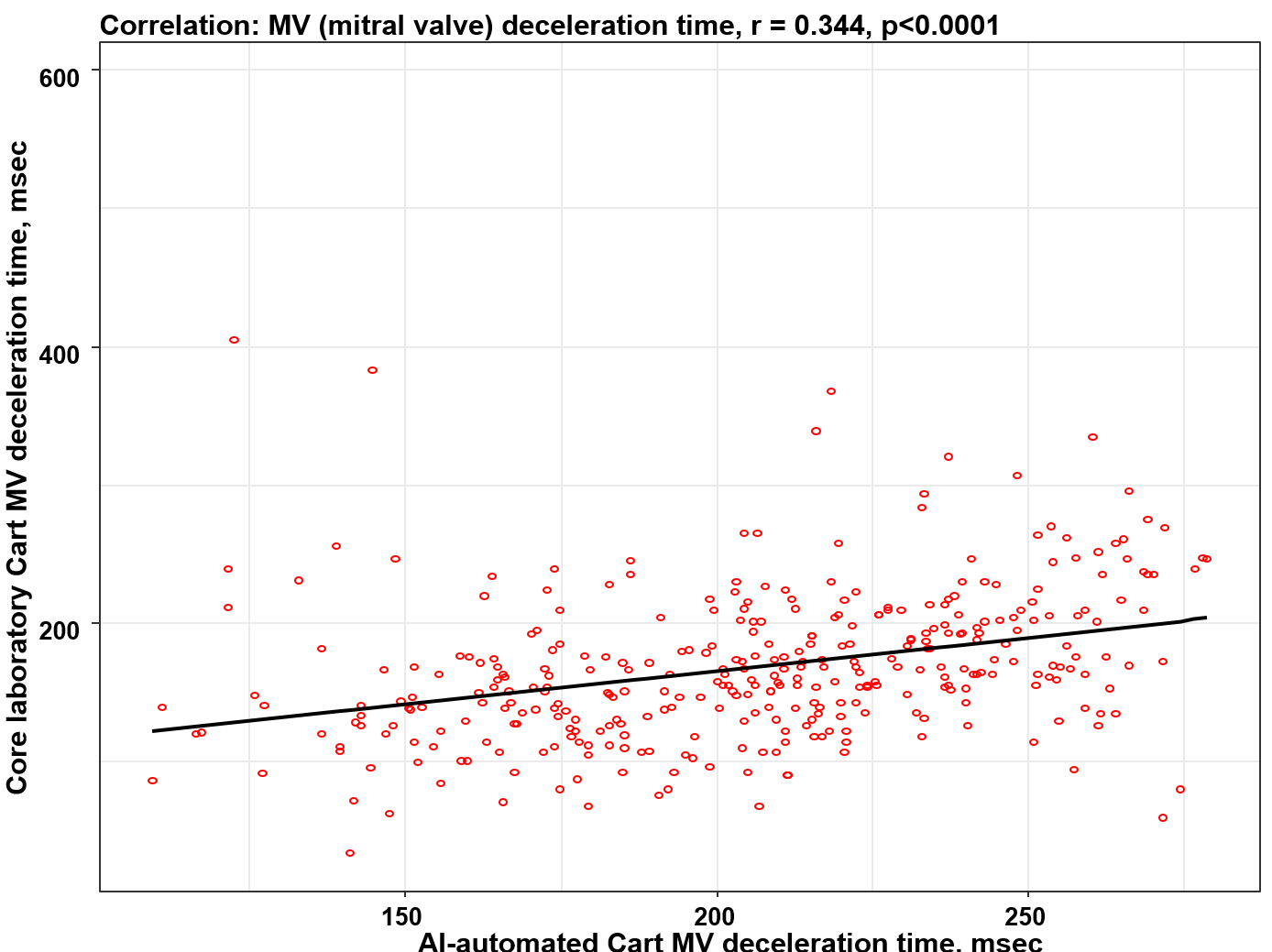


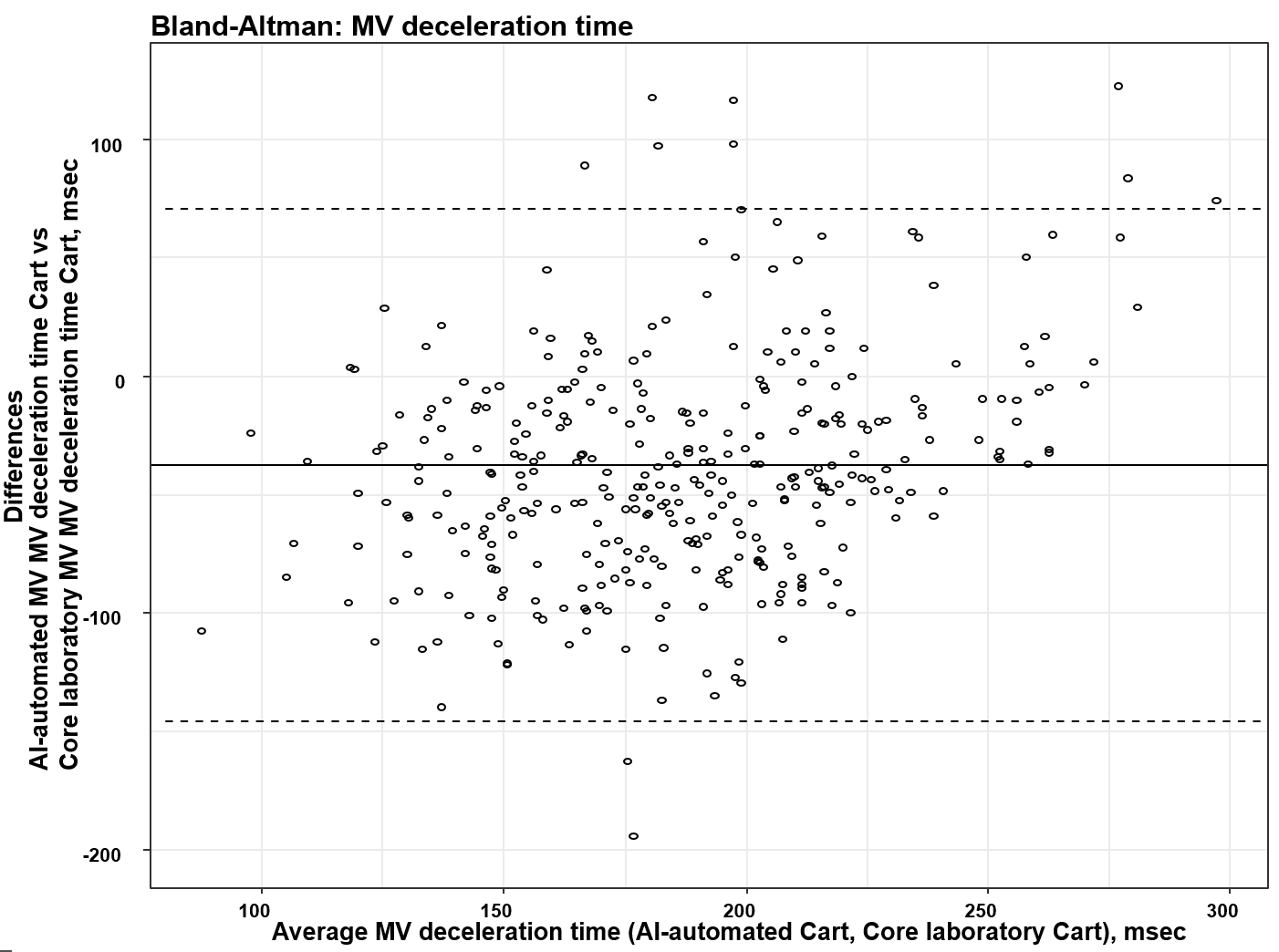


**Supplementary figure S20: mitral valve E/e’ correlation and Bland-Altman plot**


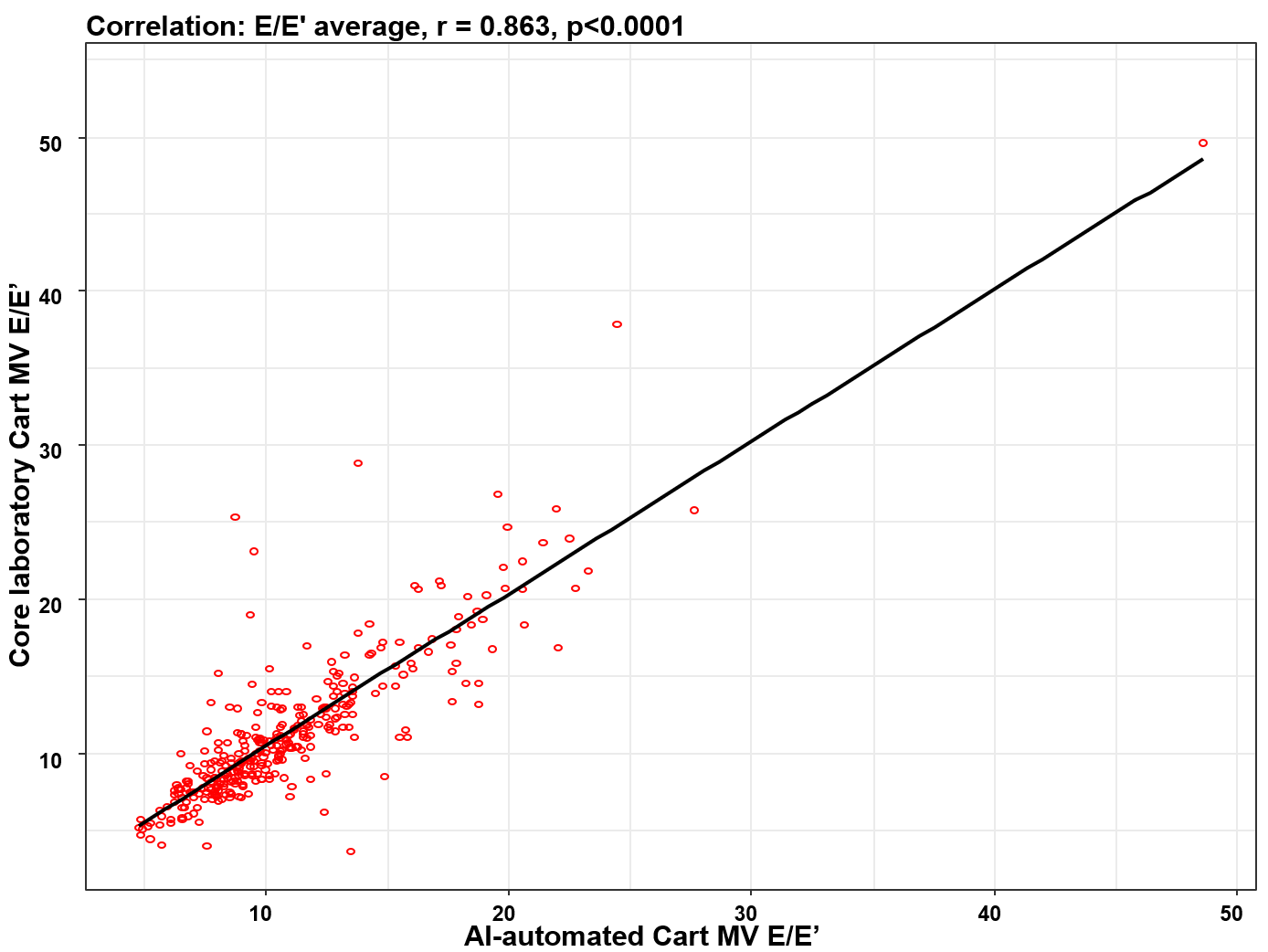


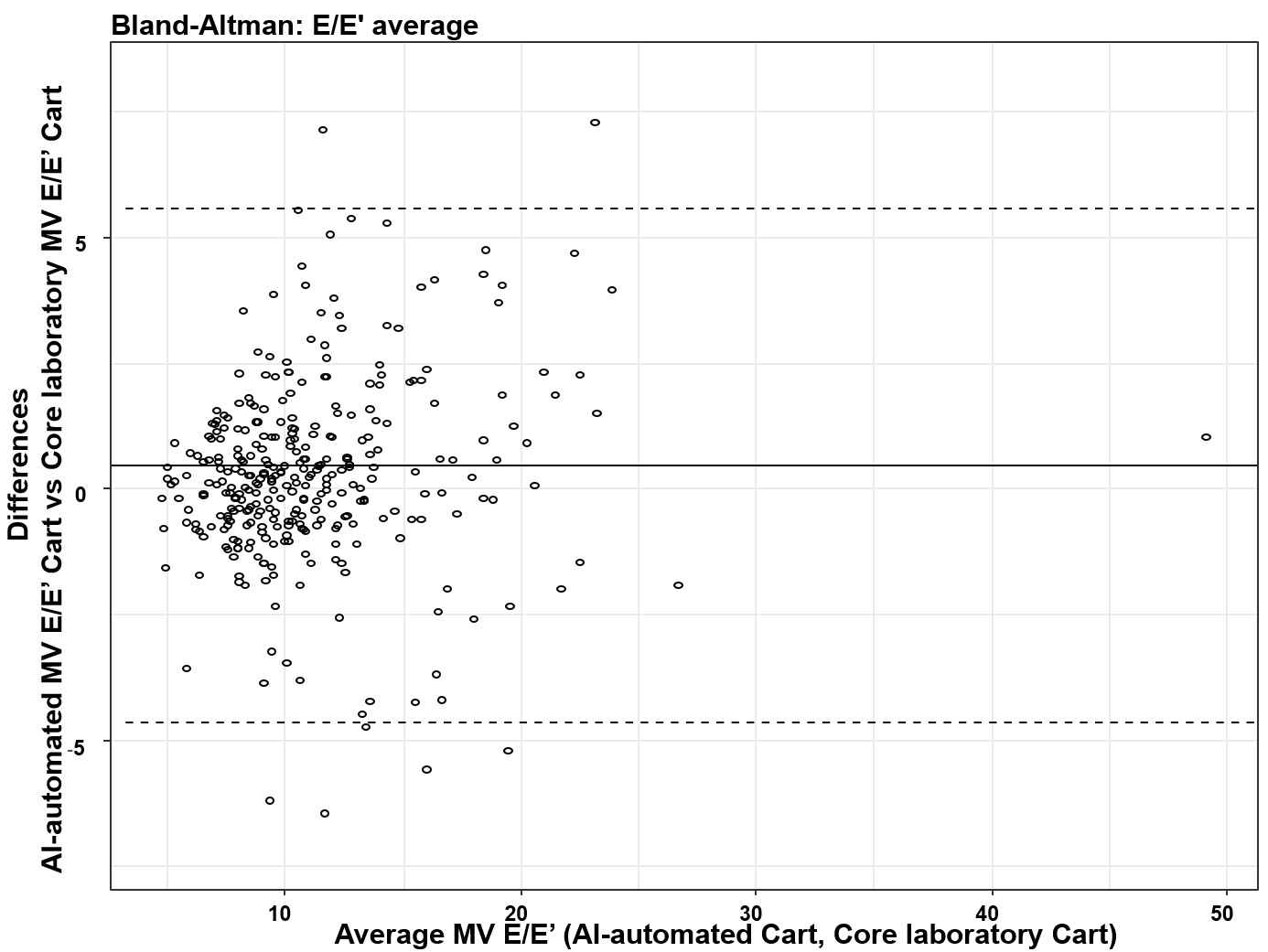


**Supplementary figure S21: Tricuspid valve peak velocity correlation and Bland-Altman plot**


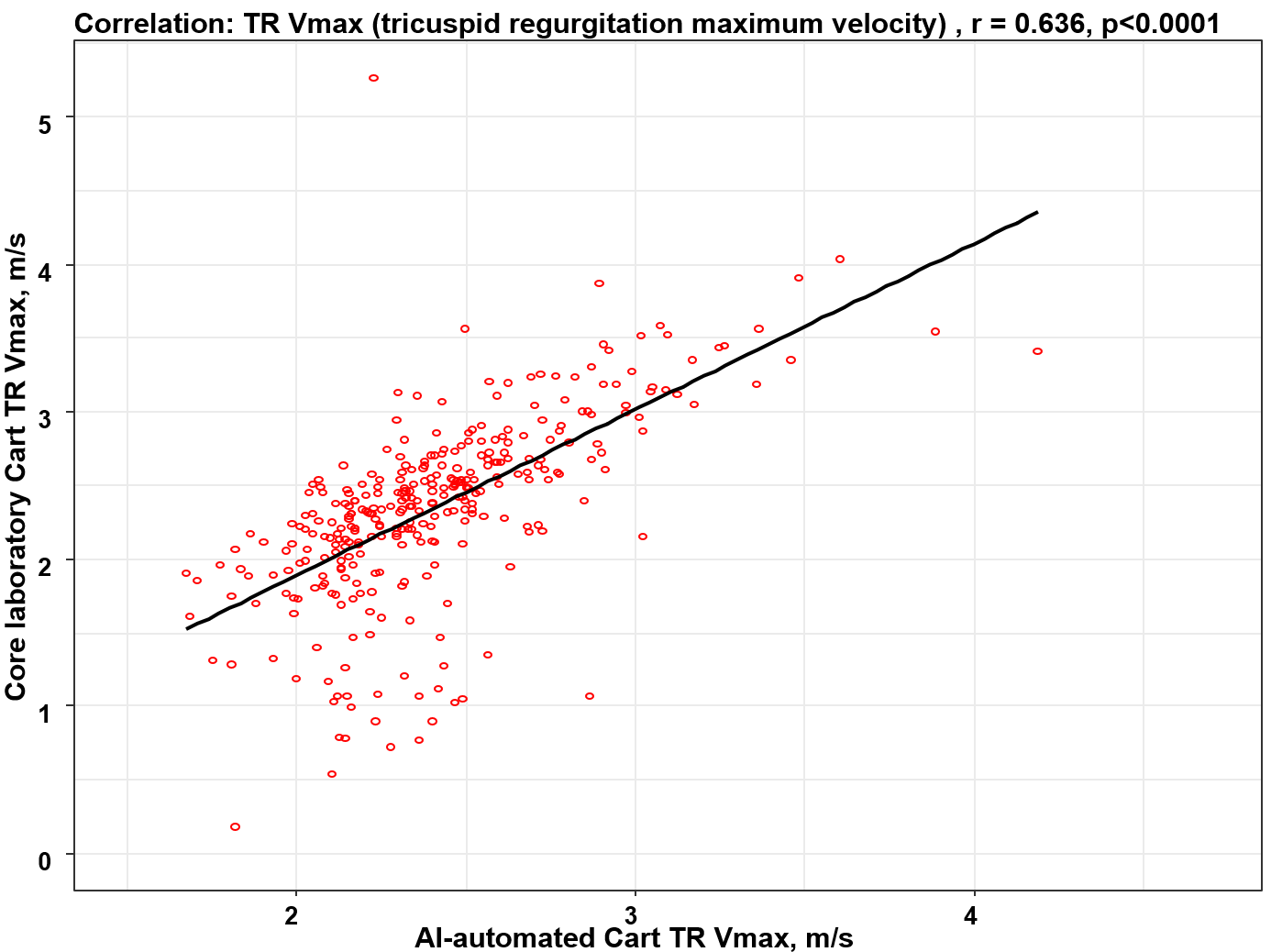


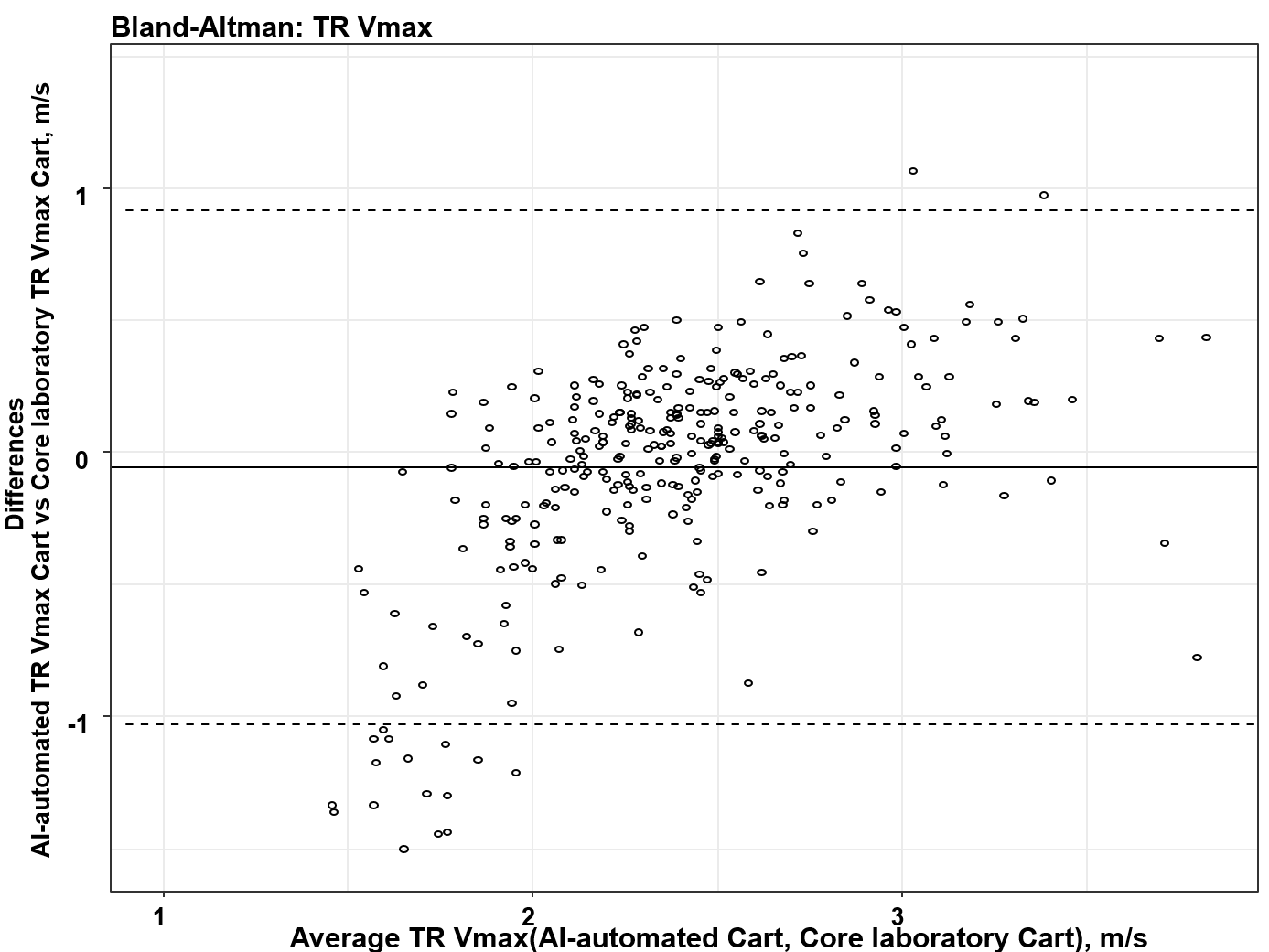

Supplement: Supplementary file 1 — Appendix S1. Supporting Information. [file EJHF-27-3401-s001.docx]
